# (−)-Oleuropein as a Novel Metastatic Castration-Resistant Prostate Cancer Progression and Recurrence Suppressor via Targeting PCSK9-LDLR Axis

**DOI:** 10.3390/nu17091445

**Published:** 2025-04-25

**Authors:** Nehal A. Ahmed, Mohamed M. Mohyeldin, Hassan Y. Ebrahim, Oliver C. McGehee, Md Towhidul Islam Tarun, Khalid A. El Sayed

**Affiliations:** 1 School of Basic Pharmaceutical and Toxicological Sciences, College of Pharmacy, University of Louisiana at Monroe, 1800 Bienville Drive, Monroe, LA 71201, USA; atefkhaledahmedabdn@warhawks.ulm.edu (N.A.A.); hebrahim@vcom.edu (H.Y.E.); mcgeheoc@warhawks.ulm.edu (O.C.M.); tarunmt@warhawks.ulm.edu (M.T.I.T.); 2 Department of Pharmacognosy, Faculty of Pharmacy, Alexandria University, Alexandria 21521, Egypt; mohamed.mohyeldin@alexu.edu.eg

**Keywords:** metastatic castration-resistant prostate cancer, oleuropein, olive phenolics, PCSK9-LDLR, protein–protein interaction, recurrence

## Abstract

**Background/Objectives**: Prostate cancer (PC) is among the most common malignancy in men. Several newly diagnosed patients have a locally advanced disease and distant metastasis at the initial diagnosis time. Castration-resistant PC (CRPC) patients have 100% recurrence incidence despite completing a therapeutic regimen, leading to high mortality. Androgen deprivation therapy and androgen inhibitors are initially effective, but resistance is inevitably developed. Epidemiological studies indicated that the Mediterranean diet, with high olive phenolic contents, is associated with a lower incidence of certain malignancies. This study aims at exploring the mCRPC progression and recurrence-suppressive and molecular effects of the major olive leaf phenolic glucoside (−)-oleuropein (OLE). **Results**: OLE downregulated the levels of proprotein convertase subtlisin/klexin type 9 (PCSK9) and normalized the low-density lipoprotein receptor (LDLR) in PC cells in vitro. Thus, a PCSK9-LDLR protein–protein interaction (PPI) in silico model was generated and used to assess OLE and its aglycone (OA) ability to bind at PCSK9 and thereby interfere with PCSK9-LDLR PPI. OLE perfectly filled the PCSK9 interface versus OA. Both OLE and OA showed virtual potential to interfere with PCSK9-LDLR PPI. OLE showed modest in vitro viability, migration, and clonogenicity suppressive effects on diverse human PC cell lines. OLE effectively suppressed mCRPC progression and recurrence in a nude mouse xenograft model. RNA-sequencing results proved the *PCSK1*, *PCSK2*, and *PCSK9* downregulation in OLE-treated recurrent tumors versus vehicle control. **Conclusions**: Oleuropein is a novel lead useful for the control of mCRPC progression and the prevention of its recurrence via targeting PCSK9 expression and PPI with LDLR.

## 1. Introduction

Prostate cancer (PC) ranks as the second most common malignancy diagnosed among men in the US, after lung cancer. According to recent cancer statistics, 313,780 estimated new cases and 35,770 deaths are expected in 2025 in the US [1]. PC progression is a multistep process, starting with prostatic intraepithelial neoplasia, advancing to localized castration-sensitive PC (CSPC), progressing to castration-resistant PC (CRPC) by losing the androgen receptor (AR) dependency, then developing into advanced mCRPC with local invasion and metastatic lesions, and finally exerting neuroendocrine transdifferentiation, driven by neuronal transcription factors to the poorly treatable neuroendocrine PC phenotype [2]. Androgen dependency/addiction is a hallmark of primary PC, leading to an initial positive response to androgen deprivation (ADT) and androgen receptor (AR)-targeted therapies. ADT involves lowering the levels of testosterone to the castrate level (<5 ng/dL), while AR pathway inhibitors (APIs) block the AR oncogenic signaling pathway. The majority of men with advanced PC eventually develop resistance to castration and are classified as CRPC, which continues to progress clinically, radiographically, or biochemically, despite serum testosterone levels being lower than castration levels [3]. Several approved systemic therapies include a combination of cytotoxic chemotherapies such as docetaxel and cabazitaxel, and second-generation APIs like enzalutamide, the CYP17A (17α-hydroxylase) inhibitor abiraterone acetate, and the radioisotope radium-223 [4]. While these therapies initially provide effective disease control, the majority of PC patients inevitably develop acquired resistance during their treatment course [3].

The low-density lipoprotein receptor (LDLR) is a cell membrane glycoprotein that plays an important role in cholesterol influx by binding plasma LDL particles and acting as a lipoprotein transporter, thus lowering systemic cholesterol levels [5].

The proprotein convertase subtilisin/kexin type 9 (PCSK9) is a 75 kDa serine protease glycoprotein, comprised of 629 amino acid residues [6,7,8,9,10,11]. It is the ninth member of the proprotein convertase family, which is mainly expressed in the liver along with minor amounts produced in intestinal tract, kidney, brain, heart, and the blood vessels [6,7,8]. It had been previously reported that PCSK9 plays a critical role in the regulation of cholesterol homeostasis. PCSK9 promotes LDLR degradation by enhancing its lysosomal degradation, instead of normal recycling back to the cell surface [9]. PCSK9 expression had been reported in different cancer types [10]. PCSK9 plays a crucial oncogenic role in various cancer-related processes, including cell proliferation and survival, invasion, metastasis, resistance to radiation therapy, and tumor immune response [11]. Previous studies have demonstrated that patients with high levels of tumoral *PCSK9* mRNA expression levels have poorer overall survival compared to those with lower expression levels across different patient cohorts [12]. A recent study showed that PCSK9 is overexpressed in colon cancer tissues and correlated with a worse tumor pathological grade [8]. Genetic variants of PCSK9 that increase LDL levels were associated with a higher risk of breast cancer, while LDL-lowering variants, resembling the effects of PCSK9 inhibitors, showed a significant correlation with a reduced risk of developing breast cancer [13]. PCSK9 produced by tumors plays a pivotal role in the development of melanoma by exerting systemic effects on the immune system, facilitating immune evasion by the melanoma cells [14]. PCSK9 overexpression boosted gastric cancer metastasis and repressed apoptosis by supporting the MAPK signaling pathway through HSP70 upregulation [15]. PCSK9 proved to drive CRPC progression and recurrence [16,17]. A high-fat diet (HFD, 11% fat content) was found to double the PCSK9 expression level in mCRPC compared to a regular chow diet (5% fat content) [16]. A higher PCSK9 immunohistoscore was observed in early stage human PC patient tissue microarray data [16]. Genetically proxied PCSK9 suppression was correlated with reduced total and early-onset PC risks, suggesting the unique oncogenic role for PCSK9 in PC [18].

The Mediterranean diet, rich in olive phenolics, is well-reputed for better longevity, lower cardiovascular-related morbidity, and other numerous positive health outcomes, including reduced incidence to certain malignancies [19,20]. (−)-Oleuropein (OLE), a major olive leaf extract phenolic secoiridoid glucoside, is a bitter-tasting ester of hydroxytyrosol with an oleosidic skeleton (Supplementary Figure S1). The oleosidic moiety is common in secoiridoid glucosides of the family Oleaceae [21]. OLE is the most plentiful phenolic ingredient, occurring in olive leaves, seeds, pulps, and the peel of unripe olives. OLE showed promising anticancer activities against multiple cancer types. OLE enhanced cytotoxicity and upregulated peroxiredoxin in luminal A MCF-7 breast cancer cells [18,22]. Combining OLE with paclitaxel at low doses showed synergistic effects on MCF-7 cells [23]. OLE effectively reprogrammed myeloid cells and synergized with anti-PD-1 therapies against lung and colon cancers [24]. OLE exhibited a remarkable apoptosis-inducing tendency and reversed cisplatin resistance in A2780 ovarian cancer cells [25]. OLE suppressed breast and colorectal cancers through modulating COX-2, NF-κB, ROS, and PTP1B [26]. The present study validates OLE as an effective mCRPC progression and recurrence suppressor lead via targeting PCSK9 expression and PPI with LDLR.

## 2. Materials and Methods

### 2.1. Molecular Modeling

#### 2.1.1. Computational Protein Structure Acquisition and Preparation

In silico experiments were carried out using the Schrodinger molecular modeling software package (New York, NY, USA) installed on an iMac 24-inch workstation with an M3 chip, 8-core CPU, 10-core GPU, 16-core Neural Engine processor, and 16 GB RAM with a Retina 4.5 K display (Apple, Cupertino, CA, USA). The X-ray crystal structure of PCSK9 in complex with the epidermal growth factor-like repeat A (EGF-A) domain of the LDLR, was retrieved from the RCSB Protein Data Bank (PDB) in pdb format (PDB ID: 3BPS). The crystal structure 3BPS was selected because its X-ray diffraction was run at a high resolution of 2.41 Å, which would decrease the possibilities of false positive results because of conformational variations. PCSK9 and EGF-A were prepared for protein–protein docking using the PrepWiz module in Schrodinger suite. Each protein was preprocessed by assigning bond orders, adding hydrogens, and creating disulfide bridges as well as zero-order bonds to metals. Hydrogen bonding networks were then optimized using PROPKA (Jensen Research Group, Copenhagen, Denmark) and the terminal and loop ends for each protein were capped with neutral amine or aldehyde groups at physiological pH ± 2. Finally, energy minimization was implemented for PCSK9 and EGF-A using an Optimized Potentials for Liquid Simulation (OPLS4) force field with excellent RMSD values of 0.06 and 0.12, respectively [27].

#### 2.1.2. Protein–Protein Docking Studies

Molecular modeling was used to assess whether OLE will interfere with PCSK9-LDLR binding. A protein–protein docking study was used to generate a PCSK9-LDLR PPI model. The PPI docking panel of the BioLuminate module (BioLuminate version 5.6.138, 2024-3, Schrodinger, New York, NY, USA) was used to dock the LDLR EGF-A domain as a ligand protein to the PCSK9 as a receptor protein in a standard mode. The ligand LDLR was rotated into 70,000 different orientations with respect to the PCSK9 receptor protein, and each of the ligand orientations was translated to find the best docking score. The top 1000 rotations were clustered using the RMS distance between matching atoms in each pair of rotated structures. The structure that was taken from each cluster was the one with the most neighbors in the cluster. After docking experiments have been concluded, an additional PPI analysis experiment was carried out for each docking output to identify important residues for the PCSK9-LDLR interaction using the protein interaction analysis panel of the Piper interface of the BioLuminate module (BioLuminate version 5.6.138, 2024-3, Schrodinger, New York, NY, USA). Briefly, the PPI analysis panel analyzed the interactions at the interface of PCSK9 and the EGF-A domain of LDLR, defined as two sets of chains, and located residues in the second set of chains that are within a given 4.0 Å distance of residues in the first set of chains. Finally, the analysis presented counts of hydrogen bonds, salt bridges, disulfide bonds, π-π stacking interactions, as well as van der Waals clashes, and reported the van der Waals surface complementarity and buried solvent-accessible surface area. The results of the protein interaction analysis are provided in Supplementary Table S1.

#### 2.1.3. Protein Structure Alignment

Protein alignment was performed between the predicted PCSK9-LDLR model and that of the crystal structure 3BPS using the protein structure alignment panel of BioLuminate module (BioLuminate version 5.6.138, 2024-3, Schrodinger, New York, NY, USA). This panel enabled the superimposition of both PCSK9-LDLR complexes into a common frame with an algorithm that attempted to align secondary structure elements. The PCSK9 protein backbone from 3BPS was used as a reference residue subset, whose frame was considered for alignment. After the completion of the alignment calculation, structures were overlaid in the same frame of reference. In addition, the aligned residues were listed in a separate text file and an alignment score and RMSD value were calculated. The RMSD was determined from aligned C-α atoms of protein residues. The alignment score provided a quantitative measure of alignment quality and it was calculated using an algorithm established earlier [28]. Score thresholds have been described as guidelines for interpreting the degree of structural similarity between proteins. A score below 0.6 indicated a good structural alignment, suggesting significant structural resemblance between the overlaid proteins. Meanwhile, scores exceeding 0.7 suggested poor alignment, implying that compared structures demonstrated substantial differences. In some cases, the algorithm may fail to calculate an alignment score altogether. This typically occurs when proteins are too dissimilar to be meaningfully aligned.

#### 2.1.4. Detection of Druggable Binding Sites Using SiteMap

The SiteMap panel of the Maestro interface (Maestro version 14.1.138, 2024-3, Schrodinger, New York, NY, USA) was used to find, visualize, and characterize possible PCSK9 protein binding sites, which are amenable to accommodate small molecules. This mapping system also provided a quantitative method to evaluate and rank potential binding sites on proteins [29]. Such an evaluation employed site-point groups and energetic properties of grid points to assess the binding sites. SiteMap generated several properties for analyzing protein binding sites, including the volume and size of each possible pocket, with the overall SiteScore being the most significant as a valuable predictive tool. This score has demonstrated effectiveness in identifying known binding sites in protein–ligand complexes, and it can be interpreted as follows: A SiteScore exceeding 1.0 indicates a highly promising site for small molecule binding. Meanwhile, a SiteScore of 0.8 serves as a critical threshold, which has been shown to effectively differentiate between sites that are likely to bind drug-like molecules and those that are less likely to represent druggable sites.

#### 2.1.5. Ligand Structure Preparation

The LigPrep module of the Maestro interface (Maestro version 14.1.138, 2024-3, Schrodinger, New York, NY, USA) was applied to create 3-dimensional structures and to search for different conformers of OLE and its aglycone (OA). Chemical structures were first sketched on the Maestro panel interface (Maestro version 14.1.138, 2024-3, Schrodinger, New York, NY, USA). The OPLS4 force field was applied to geometrically optimize compound structures and to compute partial atomic charges [27]. Possible poses with different steric features (up to 32) were generated for each compound for subsequent molecular docking studies.

#### 2.1.6. Grid Preparation

The prepared protein structure of PCSK9 was used to create receptor energy grids using the receptor grid generation module of the Maestro panel interface (Maestro version 14.1.138, Schrodinger, New York, NY, USA). The module was used to locate the binding site by applying the default value of the protein atomic scale (1.0°A) within the cubic box centered upon the sitemap points, which defined the potential druggable site identified by SiteMap.

#### 2.1.7. Protein-Ligand Docking Studies

OLE and OA were docked using the Glide module interface (Maestro version 14.1.138, Schrodinger, New York, NY, USA) in extra-precision (XP) mode. Docking scores were generated using the Glide-Dock program’s empirical scoring functions. A second cycle of protein–protein docking was then undertaken for the EGF-A domain of the LDLR as a ligand towards PCSK9-OLE or PCSK9-OA complexes as a receptor, using the BioLuminate module (BioLuminate version 5.6.138, 2024-3, Schrodinger, New York, NY, USA) in each case and applying the same parameters as shown in Section 2.1.2.

### 2.2. Chemicals, Reagents, and Antibodies

All chemicals and reagents were obtained from Sigma-Aldrich (St. Louis, MO, USA), unless specified differently. Organic solvents were purchased from VWR (Suwanee, GA, USA), dried by standard procedures, packaged under nitrogen in Sure/Seal bottles, and stored over 4 Å molecular sieves, unless otherwise indicated. All antibodies were purchased from Cell Signaling Technology (Beverly, MA, USA), except where noted. Cell culture reagents were purchased from Life Sciences (Carlsbad, CA, USA).

### 2.3. Cell Lines and Culture Conditions

The human immortalized non-tumorigenic prostate epithelial cell RWPE-1, and human PC cell lines LNCaP (hormone-dependent), DU-145, and PC-3 (hormone-independent) were obtained from the American Type Culture Collection (ATCC, Manassas, VA, USA). The CWR-R1ca (metastatic castration-resistant) cell line was purchased from Millipore/Sigma (Burlington, MA, USA). PC cells were cultured in the Roswell Park Memorial Institute (RPMI-1640), supplemented with 10% fetal bovine serum (FBS), penicillin G (100 U/mL), and streptomycin (100 ng/mL). All cells were maintained in a humidified incubator at 37 °C with 5% CO_2_. The sub-culturing of cells started by washing with Ca^2+^- and Mg^2+^-free phosphate-buffered saline (PBS) and incubating in 0.05% trypsin containing 0.02% Ethylenediaminetetraacetic acid (EDTA) in PBS for 4–7 min at 37 °C.

### 2.4. Oleuropein Extraction and Purification

Oleuropein (OLE) was extracted from dried olive tree samples, generously offered by Florida Olive Systems (DeLand, FL, USA), or purchased olive leaf extract capsules, 50% standardized oleuropein content (Nusapure^®^, Amazon, Seattle, WA, USA), by maceration overnight in MeOH, followed by defatting with *n*-hexanes. The extract was then adsorbed on Celite 545 and dried in a vacuum oven. Purification was achieved on a Sephadex LH-20 column (1:50 *w*/*w* ratio dry extract-stationary phase) using a careful gradient elution starting with CH_2_Cl_2_, followed by increasing amounts of MeOH up to 8% to afford 96 fractions, 2 mL each. Recognized by TLC against a standard OLE, fractions containing OLE were pooled and dried. The identification of OLE was determined by a mass spectrometric analysis on a JEOL JMS-T100LP AccuTOF LC-plus instrument (Peabody, MA, USA), and further confirmation of purity was achieved by q^1^H NMR in deuterated methanol (CD_3_OD) on a JEOL JNM-ECZL400S FT-NMR system with Delta™ processing software (version 6.3, Peabody, MA, USA), confirming > 99% OLE purity.

### 2.5. In Vitro Cell Culture Assays

#### 2.5.1. Cell Proliferation Assay

Cells were seeded into 96-well plates at a density of 1 × 10^4^ cells/well in 100 μL of culture medium, and the plates were incubated overnight at 37 °C in a 5% CO_2_ humidified incubator for cell attachment overnight. The next day, OLE was prepared as a stock solutions (10 mM) in DMSO and immediately added to culture media to prepare the final working concentrations. About 100 μL of treatment media was added, in triplicates, and cells were incubated at 37 °C for 72 h. The media were gently aspirated, and cells were rinsed with sterile PBS. About 100 μL of fresh media and 100 μL of MTT solution added were to each well and cells were incubated for an additional 3 h. Supernatants were carefully removed, and formazan crystals were dissolved in 100 μL of DMSO. The plates were incubated for 10 min before measuring the absorbance at 570 nm using a Synergy 2 microplate reader (BioTek, Winooski, VT, USA). Cell numbers were derived from a standard curve conducted at the beginning of each experiment. IC_50_ values were calculated using GraphPad Prism version 8.01 (GraphPad Software, San Diego, CA, USA).

#### 2.5.2. Wound-Healing Scratch Assay

Cells were plated in 24-well plates at a density of 5 × 10^3^ cells/well and incubated overnight to attach and differentiate at 37 °C in a 5% CO_2_ humidified incubator. Wounds were scratched in confluent monolayers using sterile 200 μL pipet tips. Cells were washed with PBS and re-incubated in 1% serum media containing various concentrations of OLE from a 10 mM stock solution in DMSO or DMSO as the vehicle control. Wounds were photographed at 0 h and monitored for wound closure up to 24 h. When wounds were about to close, the media were gently aspirated and cells were rinsed with cold PBS and fixed with ice cold MeOH, stained by Giemsa stain for 10 min, and rinsed 3× with tap water. Finally, wound images were captured, and treatments were compared with the vehicle control treatment using a Nikon Ti2-A Inverted Intelligent microscope (Nikon Instruments Inc., Melville, NY, USA). The percentages of cell migration in each treatment were calculated using the following formula: percent cell migration = [T0 − Tt − Tdmso]/[T0 − Tdmso] × 100, where T0 is the wound thickness at 0 h, Tdmso is the wound thickness in DMSO-treated control wells, and Tt is the wound thickness in treatment wells. IC_50_ values were calculated using GraphPad Prism version 8.01 (GraphPad Software).

#### 2.5.3. Colony Formation Assay

Cells were seeded in 12-well plates at a density of 1 × 10^3^ per well and incubated for 3 days until attachment and differentiation at 37 °C in a 5% CO_2_ humidified incubator. Different concentrations of OLE (5–160 μM) were prepared in 1% serum media from a 10 mM stock solution in DMSO. For each treatment, fresh media were changed every other day for a 12-day incubation period. At the end of the incubation period, media were removed, colonies were washed with cold PBS, fixed with ice-cold MeOH, stained by Giemsa stain for 10 min, and rinsed 3× with tap water. Images were captured using a digital camera and colonies were counted manually.

#### 2.5.4. Western Blot Assays

The human mCRPC CWR-R1ca cells were seeded at a density of 1 × 10^6^ cells/100 mm in a culture dish and incubated overnight to attach. Cells were treated with either OLE in 3 concentrations prepared from the 10 mM stock solution or DMSO as the vehicle control for 72 h. Cells were washed with cold PBS and collected to prepare cell lysates. Total protein contents were extracted using the RIPA lysis buffer (Thermo Fisher Scientific, Madison, WI, USA), supplemented with mammalian protease arrest (G-Biosciences, St. Louis, MO, USA) and incubated at 4 °C for 30 min. Lysates were centrifuged for 15 min at 15,000× *g* and supernatants were stored at −80 °C. Animal tissues (liver) and tumor samples were weighted, and their total protein contents were extracted in RIPA lysis buffer (Thermo Fisher Scientific, Madison, WI, USA), supplemented with mammalian protease arrest (G-Biosciences, St. Louis, MO, USA), and tissues were homogenized was using an ultrasonic homogenizer (Qsonica Sonicator, Newtown, CT, USA). Homogenates were incubated at 4 °C for 4 h, then centrifuged for 15 min at 14,000× *g*, and supernatants were stored at −80 °C. The protein concentration was determined by the Pierce BCA Protein Assay (Bio-Rad, Hercules, CA, USA). Lysates were loaded as cell lysate 10 μg, liver lysate 10 μg, and tumor lysate 15 μg. Proteins were electrophoresed on Mini-PROTEAN TGX precast polyacrylamide gels (BIO-RAD) using Tris/glycine/SDS running buffer and transferred to Immuno-Blot PVDF membranes (BIO-RAD). Blotted membranes were blocked with 5% BSA (Cell Signaling Technology, Beverly, MA, USA) in TBST (10 mM Tris-HCl, 150 mM NaCl, 0.1% Tween-20) for 2 h with gentle agitation at rt. Immunoblots were incubated overnight at 4 °C with appropriate primary antibodies (Cell Signaling Technology). After incubation, membranes were washed 5 times with TBST and then probed with HRP-labeled secondary antibodies (Cell Signaling Technology) for 1 h with agitation at rt, followed by rinsing 5 times with TBST. Proteins were detected using the ChemiDoc XRS chemiluminescent gel imaging system and analyzed using Image Lab software (Version 5.2.1, Bio-RAD, Hercules, CA, USA).

### 2.6. In Vivo Studies

#### 2.6.1. Animal Model and Treatment Modes

Male athymic nude mice (Foxn^1nu^/Foxn^1+^, aged 5–6 weeks) were purchased from Envigo (Indianapolis, IN, USA). The mice were maintained at the University of Louisiana at the Monroe (ULM) animal facility. Animal were housed in filter-top cages with Alpha-Dri bedding in a clean environment. The cages were placed on ventilated racks equipped with high-efficiency particulate air (HEPA) filtration. Environmental conditions were controlled at 25 °C, 55–65% relative humidity, with a 12 h light/dark cycle. Mice received a high-fat Teklad S-2335 Mouse Breeder Sterilizable Diet (total fat 11.4%, crude proteins 17.2%, and carbohydrates 45.2%). Animal experiments were approved by the Institutional Animal Care and Use Committee (IACUC), ULM, protocol numbers 19NOV-KES-02 and 23MAR-KES-01. All experiments were conducted in a strict accordance with the NIH-guided good animal practices.

#### 2.6.2. Tumor Progression Model

For the mCRPC CWR-R1ca-luciferase labeled model, approximately 5 × 10^6^ cells were xenografted subcutaneously into the mouse suprascapular region [30]. When the tumors became palpable with an average tumor volume reaching 70–100 mm^3^, nearly 21 days after xenografting, mice were randomly assigned into the vehicle control (sterile PBS) and OLE 75 mg/kg, ip, 3×/week treatment groups (*n* = 5, each). Treatments continued for 8 weeks. Biweekly live isoflurane-anesthetized mice bioluminescence images were captured using the IVIS Lumina Series III (PerkinElmer, Waltham, MA, USA) imaging system after intraperitoneal (ip) injection with D-luciferin (XenoLight D-luciferin K^+^ salt bioluminescent substrate, Perkin Elmer, Waltham, MA, USA) at a dose of 150 mg/kg per animal in sterile PBS. The photons were emitted from luciferase-expressing cells within the animal body, transmitted through the tissue, and quantified as photons/second using the Living Image software program (Version 4.7.3, PerkinElmer, Waltham, MA, USA). Images representing light intensity (blue, least intense, and red, most intense) were generated [16,31]. The tumor volume (V) in each mouse was monitored and calculated by the formula V = (L × W^2^)/2, where L is the tumor length and W is the width. The animals’ health status was observed routinely by carefully monitoring animals’ body weight changes or any signs of altered behavioral or motor ability. Tumors were then surgically excised after animals were isoflurane-anesthetized, and tumor samples were snap frozen in liquid nitrogen or cryogenically preserved in RNA-later and stored at −80 °C until total protein extraction for the Western blot and RNA-sequencing analysis.

#### 2.6.3. Tumor Recurrence Model

Mice that recovered from the primary tumor excision surgery in the progression model were used to study the OLE ability to prevent the mCRPC recurrence. Animals were monitored visually and bioluminescence imaged biweekly for locoregional or distant recurrences. Mice continued receiving the same dosage regimen: OLE-treated group (75 mg/kg, 3×/week, ip) or the sterile PBS VC for an additional 8 weeks. At the end of the study, mice were sacrificed and bioluminescence images of each mouse’s whole body and collected organs (liver, lung, brain, bone, and kidney) were captured using the PerkinElmer IVIS Lumina Series III (Waltham, MA, USA) imaging system to visualize locoregional and distant tumor recurrences.

### 2.7. RNA Extraction

About 50–90 mg of each excised tumors, stored in RNAlater™ solution (Invitrogen, by Thermo Fisher Scientific, Waltham, MA, USA), were mixed with 1 mL TRizol (Invitrogen-Thermo Fisher Scientific, Waltham, MA, USA) in RNAase/DNAase free Eppendorf tubes and mechanically homogenized using the MISONIX sonicator (Division of QSonica LLC, Newtown, CT, USA). After 4 h of sample incubation on ice, 200 µL of molecular grade CHCl_3_ (Thermo Fisher Scientific, Waltham, MA, USA) was added to each tube and incubated at rt for 3 min. Samples were vortexed for 30 s, then kept in ice for 15 min for phase separation. The white layer was collected and then centrifuged at 4 °C, 12,000× *g* for 15 min. The transparent aqueous supernatant was carefully transferred to clear microtubes; then, 500 µL of molecular grade isopropanol (Thermo Fisher Scientific, Waltham, MA, USA) was added, and samples were incubated on ice for 30 min. Samples were centrifuged at 4 °C, 12,000× *g* for 10 min and the isopropanol was cautiously decanted. RNA pellets were dissolved in 70% molecular grade ethanol and centrifuged at 4 °C, 7500× *g* for 5 min, and washed twice. The supernatant was discarded and the collected RNA pellets were left to air dry for 2–3 min. The RNA pellets were dissolved in 30–50 µL nuclease free water (VWR International, LLC, Radnor, PA, USA); then, the RNA concentration was quantified by a NanoDrop One microvolume UV-visible spectrophotometer (Thermo Fisher Scientific, Waltham, MA, USA). RNA samples at concentrations ranging from 200 ng to 1 ug of total RNA were utilized for RNA-sequencing.

### 2.8. RNA-Seq Data Processing

RNA-sequencing was performed at a strand-specific 100 cycle paired-end resolution, in an Illumina NovaSeq 6000 sequencing machine (Illumina, San Diego, CA, USA). The 26 samples were multiplexed in two lanes of a flow-cell, with results between 48.71 and 40.04 million reads per sample. The read quality was assessed using the FastQC software, version 0.12.0 [32]. On average, the Phred quality score measured in the Phred quality scale was above 30 for all samples. As expected for RNA-sequencing data, QC did not reveal the presence of adapters that required trimming in the sequenced reads. The reads were mapped to the joint human (GRCh38) and mouse (GRCm39) genomes using the STAR software, version 2.7.11b. Almost 91% of the sequenced reads were mapped to the combined genome, with results between 42.9 and 36.0 million mapped reads per sample, of which on average 80.4% were uniquely mapped reads. Transcript abundance estimates were calculated using the FeatureCounts software, v2.22.1 [33]. Expression normalization and differential gene expression calculations were performed using the DESeq2 software, v1.49.0, to identify statistically significant differentially expressed genes. The significance *p*-values were adjusted for testing multiple hypotheses by the Benjamini and Hochberg method, establishing a false discovery rate (FDR) for each gene [34].

### 2.9. Statistical Analysis

The data analysis was performed using GraphPad Prism software, version 8.0.2. (La Jolla, CA, USA). Results were presented as mean ± standard deviation (SD) for continuous variables. Differences among various treatment and control groups in the animal study were determined by the paired Student’s *t*-test, and for the *p*-value implications, a difference of * *p* < 0.05 was considered statistically significant (* *p* < 0.05, ** *p* < 0.01, and *** *p* < 0.001).

## 3. Results

### 3.1. In Silico Prediction of Protein–Protein Binding Interactions Between PCSK9 and LDLR

The modeling study of the PCSK9-LDLR complex structure revealed that both proteins interact through a shallow contact interface between the catalytic domain of PCSK9 and the EGF-A domain in LDLR, together with a central hydrophobic region and surrounding polar amino acids capable of forming putative salt bridges, contributing to the binding affinity (Supplementary Figure S2). Four key interactions between PCSK9 and LDLR were determined:

i. Two hydrogen bonding interactions, one salt bridge between Arg194 in PCSK9 and Asp310 in LDLR (Supplementary Figure S2A), and several interaction clashes (van der Waals interactions) between PCSK9 Arg194 and Asp310 and LDLR Asn295 (Supplementary Figure S2B).

ii. One hydrogen bonding interaction between Asp238 in PCSK9 and Asn295 in LDLR (Supplementary Figure S2C) and several interaction clashes (van der Waals interactions) between Asp238 in PCSK9 with Asn295 in LDLR (Supplementary Figure S2D).

iii. Several interaction clashes (van der Waals interactions) between Asp374 in PCSK9 and His306 and Val307 in LDLR (Supplementary Figure S2E).

iv. Two hydrogen bonding interactions between Phe379 in the catalytic domain of PCSK9 via its backbone carbonyl with Asn301 and Cys308 in LDLR (Supplementary Figure S2F), in addition to several good clashes (van der Waals interactions) between Phe379 in PCSK9, via its backbone carbonyl and side chain, with Asn295, Cys297, Asn301, Val307, and Cys308 in LDLR (Supplementary Figure S2G).

**Figure 1:**
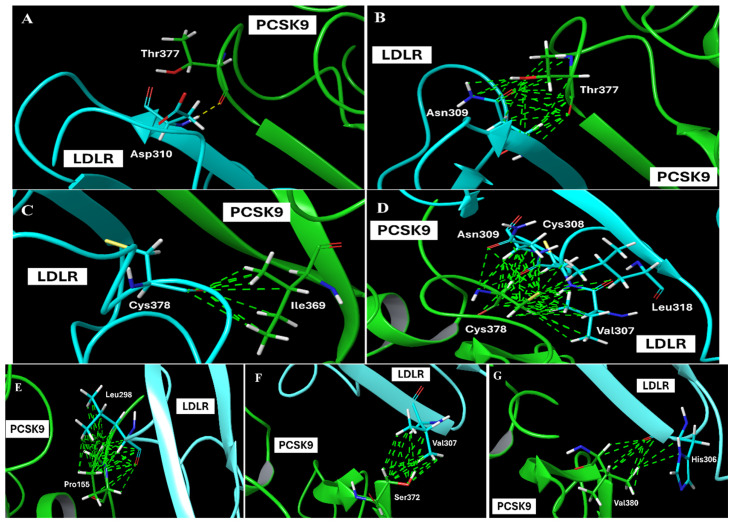
Favorable predicted molecular modeling PCSK9-LDLR interactions at various sites (**A**–**G**). Hydrogen bonds are shown as yellow dotted lines, amino acid residues interacting on the PCSK9 interface and LDLR are shown in green.

**Figure 2:**
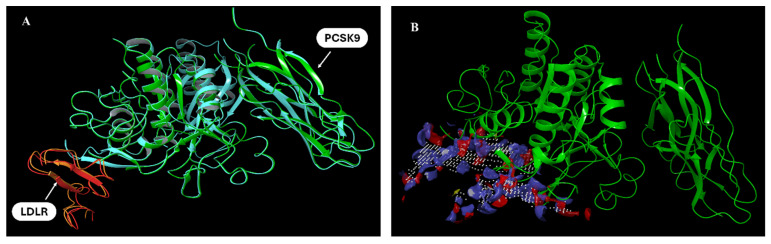
PCSK9-LDLR generated model protein alignment. (**A**) Overlay of docked pose of PCSK9 (green)-LDLR (orange) using the PDB available crystal structure 3BPS PCSK9 (blue)-LDLR (red). (**B**) Possible binding site detection at PCSK9 using SiteMap.

The favorable PCSK9-LDLR interactions (Figure 1) generated in our model are as follows: one hydrogen bonding interaction between PCSK9–Thr-377 and LDLR EGF-A–Asp-310, a Ca^++^-coordinating residue, along with several good clashes (van der Waals interactions) with EGF-A–Asn-309 (Figure 1A,B). Ile369 and Cys378 contributed to the PCSK9 hydrophobic face. Importantly, Cys378 contacts Leu318 of LDLR EGF-A (Figure 1C,D). The *N*-terminus residue of the PCSK9 catalytic domain Pro155 in addition to Ser372 and Val380 also contributed to binding with LDLR Leu298, Val307, and His306, respectively (Figure 1E–G). A summary of the PCSK9-LDLR complex’s important key and favorable interactions in the generated model are listed in Supplementary Table S1. The protein alignment of our generated model versus the already available PCSK9-LDLR crystal structure 3BPS showed an overlay with an alignment score of 0.007 and RMSD value of 0.408 Å. Sitemap was used to identify possible binding sites for small molecules at PCSK9 that can interfere with its interaction with LDLR (Figure 2A). The most important property generated by SiteMap is an overall SiteScore, which effectively identifies known binding sites in PCSK9 and LDLR (Figure 2B).

### 3.2. In Silico Binding of OLE and OA at the PCSK9-LDLR Interaction Interface

#### 3.2.1. Docking Trials for OLE at the PCSK9-LDLR Interface

**Figure 3:**
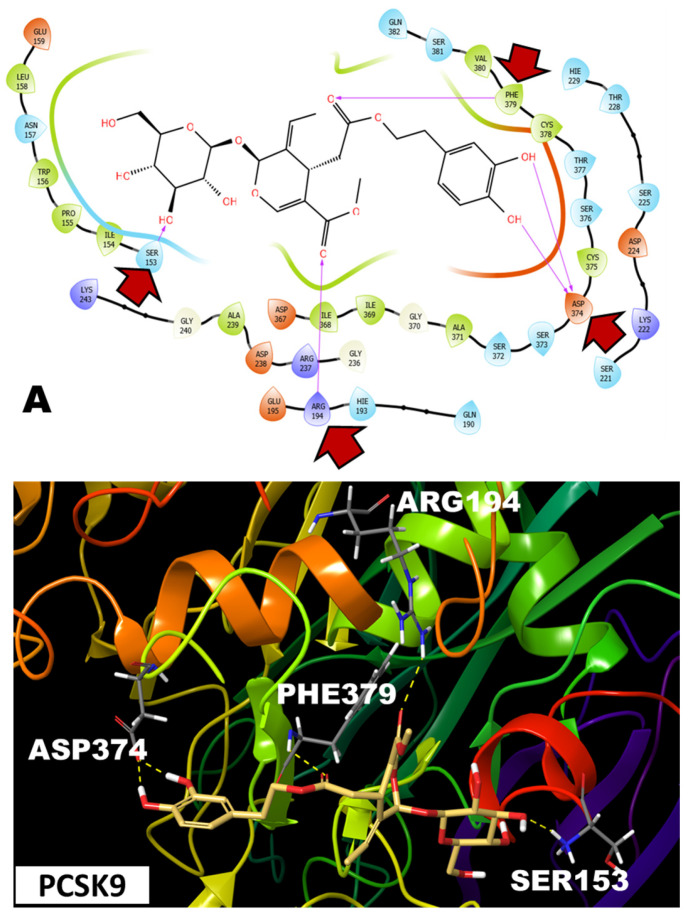

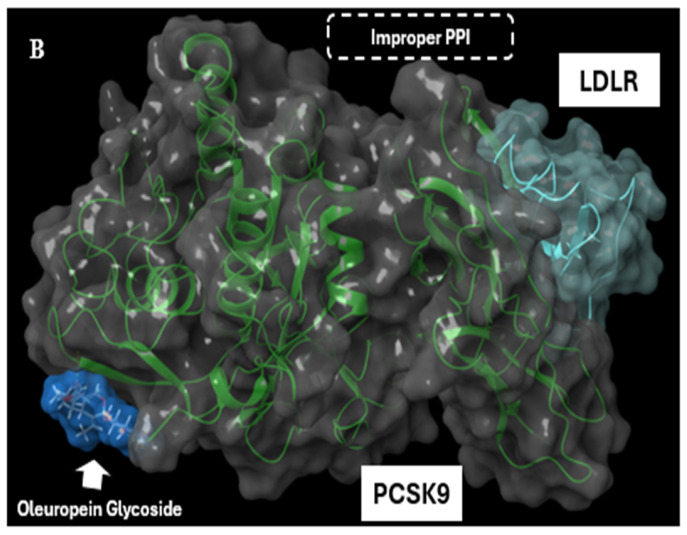
Molecular docking of OLE at the PCSK9-LDLR interface. (**A**) OLE showed favorable hydrogen bonding with the PCSK9 at the amino acid residues Asp374, Arg194, Phe379, and Ser153. Red arrows indicate critical amino acids engaged in binding interactions. (**B**) Redocking of PCSK9-OLE complex with LDLR, the presence OLE at the PCSK9 hindered the specific docking of EGF-A of LDLR at this site and resulted in an improper docking pose.

Interestingly, OLE was not only able to access the shallow PCSK9 interface with a docking score of −6.8 but also showed five key hydrogen bonding interactions with amino acids, which were involved in PCSK9-LDLR binding. Two of these hydrogen bonding interactions were through the OLE catechol moiety with Asp374, with the catechol aromatic ring exerting favorable interaction clashes with Phe379. OLE also hydrogen-bonded with the important residues Arg194 and Phe379, suggesting its promising potential to interrupt the PCSK9-LDLR binding. Finally, OLE linearly extended via its sugar moiety to interact with the *N*-terminus residue of the PCSK9 catalytic domain Ser153 (Figure 3A). Redocking the PCSK9-OLE complex with LDLR showed a completely different binding pose from that without OLE. Thus, modelling studies predicted that the OLE binding at the PCSK9 interface and its important interactions with crucial residues that might contribute to binding at LDLR can potentially hinder the specific docking of EGF-A of LDLR at this site. This resulted in an inverse docking pose, where EGF-A bounded from the other side of the PCSK9 structure, showing no interactions with the PCSK9 interface or its *N*-terminus regions involved in normal binding with LDLR (Figure 3B).

#### 3.2.2. Docking Trials for OLE Aglycone (OA) at the PCSK9-LDLR Interface

**Figure 4:**
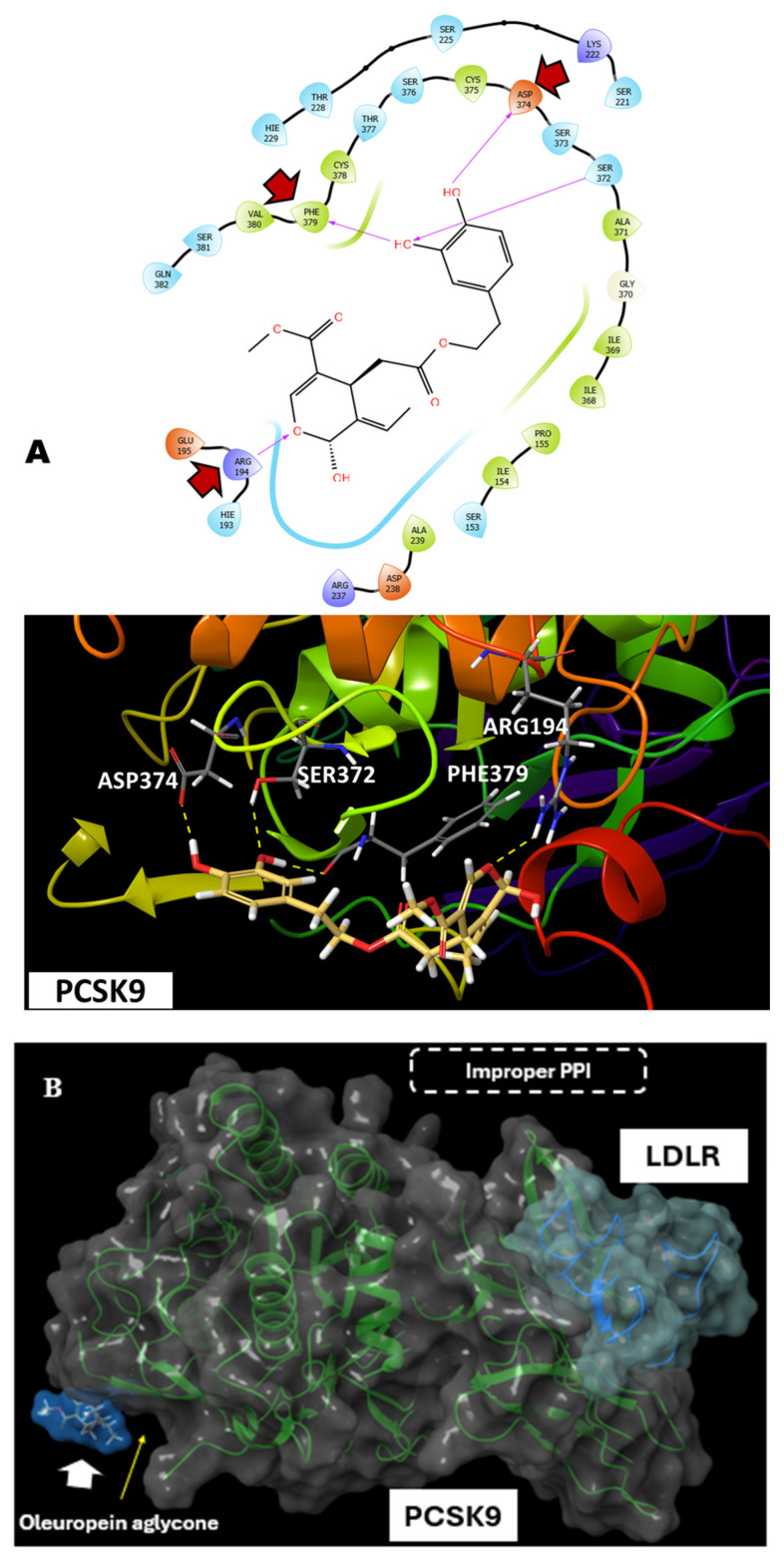
Molecular docking of OA at the PCSK9 interface. (**A**) OA showed reduced possibility to fill in the shallow interface at the PCSK9, only showing hydrogen bonding with the amino acid residues Asp374, Arg194, and Phe379. Red arrows indicate critical amino acids engaged in binding interactions. (**B**) Redocking PCSK9-OA complex with LDLR suggests that the binding of OA at PCSK9 hindered its docking ability with LDLR EGF-A.

Compared to OLE, OA showed a lower potential to occupy the PCSK9 shallow interface with a docking score of −3.2. Due to the lack of a sugar moiety, OA was not long enough to reach the *N*-terminus of PCSK9 and therefore missed the interaction with Ser153. It showed only one hydrogen bonding interaction with Asp374 and maintained the two hydrogen bond interactions with Arg194 and Phe379, which are involved in PCSK9-LDLR binding (Figure 4A). The redocking of PCSK9-OA complex with LDLR showed a completely different binding pose from that of PCSK9 without bound OA. Thus, modelling studies predict the OA potential to bind at the PCSK9 interface and its important interactions with crucial residues for LDLR binding have potential to hinder the specific docking of EGF-A of LDLR at this site. OA binding at PCSK9 can produce an inverse docking pose inaccessible to the LDLR EGF-A binding. However, unlike OLE and due to lacking the glucoside moiety, OA cannot perfectly fill the PCSK9 interface, as indicated by the yellow arrow in Figure 4B, lower left corner, suggesting that OLE can have better potential to interfere with PCSK9-LDLR PPI.

### 3.3. In Vitro Effects of OLE on the Viability of Human Non-Tumorigenic Epithelial Prostate Cells and Diverse PC Cell Lines

**Figure 5:**
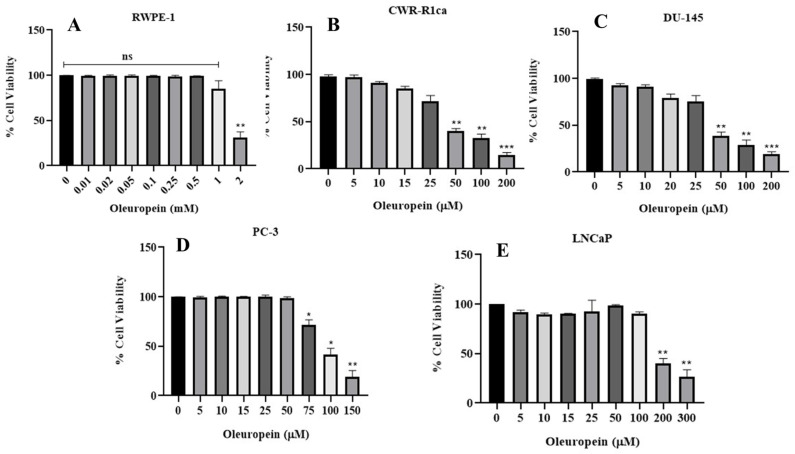
In vitro effects of OLE on the viability of the human non-tumorigenic prostate cells and diverse PC cell lines determined by MTT assay. (**A**) Effects of OLE on the viability of the human non-tumorigenic RWPE-1 cells. (**B**–**E**) Effects of OLE on the viability of the human PC cell lines CWR-R1ca, DU-145, PC-3, and LNCaP, respectively. ns: Non-statistical significance at *p* > 0.05. * Statistical significance at *p* < 0.05, ** statistical significance at *p* < 0.01, and *** statistical significance at *p* < 0.001.

The effects of OLE on the viability of the immortalized non-tumorigenic RWPE-1 epithelial prostate cells, as well as the LNCaP (androgen-dependent), PC-3, DU-145 (androgen-independent), and CWR-R1ca (mCRPC) PC cells were assessed using the MTT assay. The treatment of the human non-tumorigenic prostatic epithelial cells RWPE-1 with different OLE concentrations induced a dose-dependent cell death only at high mM levels. OLE concentrations ranging from 0.005 to 0.5 mM had no significant effect on RWPE-1 cells viability, as determined by MTT-based mitochondrial dehydrogenase metabolic activity; meanwhile, 1 and 2 mM concentrations resulted in a 15% and 69% reduction of cells’ viability, respectively (Figure 5A). On the other hand, OLE showed more potency in reducing PC cell viability with the most sensitive mCPRC CWR-R1ca cells, showing an IC_50_ of 37.6 ± 11.9 µM (Figure 5B). OLE showed moderate potency against the PC DU-145 and PC-3 cells, with IC_50_ values of 41.1± 14.6 µM and 92.4 ± 31.4 µM, respectively (Figure 5C,D). OLE exhibited the lowest reduction in cells viability on the primary hormone-dependent LNCaP cells, with an IC_50_ value of 150.8 ± 47.3 µM (Figure 5E).

### 3.4. OLE Reduced PC Cells Migration and Colony Formation

**Figure 6:**
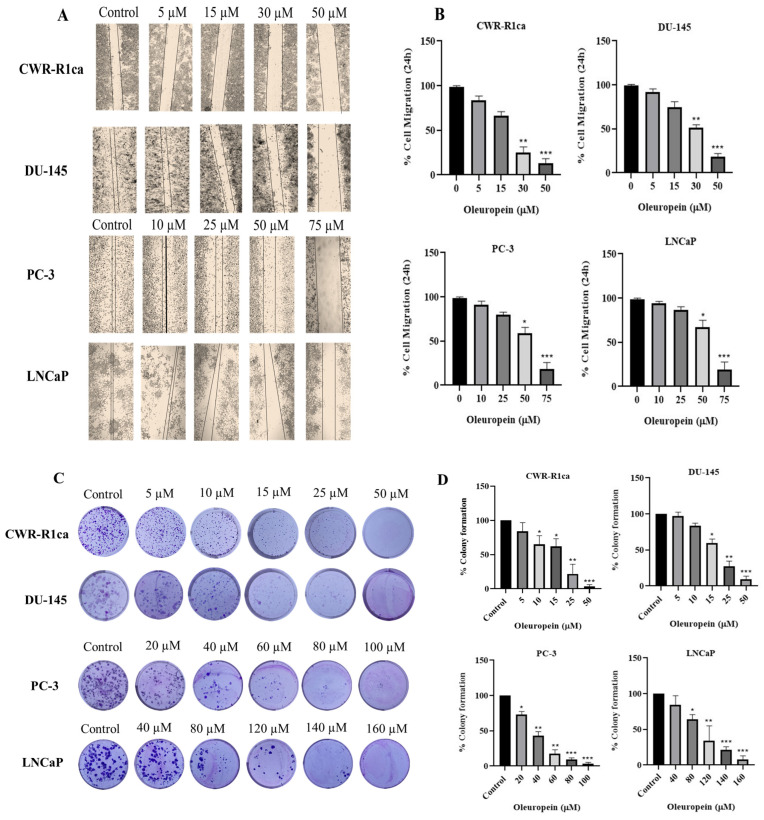
Anti-migratory and colony formation suppressive effects of OLE treatments against the migration and colonization of CWR-R1ca, DU-145, PC-3, and LNCaP PC cells. (**A**) Wound-healing assay images captured after 24 h incubation of CWR-R1ca, DU-145, PC-3, and LNCaP cells with OLE or VC. (**B**) Quantitative analysis of the percentage of migration (wound closure) with various OLE treatment doses. Vertical bars indicate the percentage of wound closure of cells after 24 h of wound scratching, calculated relative to the wound distance at 0 h in each treatment group. (**C**) Representative images of colony formation of CWR-R1ca DU-145, PC-3, and LNCaP PC cells treated with OLE treatments over 12 days and finally stained with Giemsa stain at the end of the experiment. (**D**) Quantification of OLE colony formation suppressive effects. Vertical bars indicate the percentage of colony formation relative to vehicle control. * *p* < 0.05, ** *p* < 0.01, and *** *p* < 0.001, indicate statistical significance compared to their respective vehicle-treated controls.

The migratory behavior of cancer cells is an important marker to predict the tumor’s metastatic pattern. Hence, a wound-healing assay was conducted to evaluate the anti-migratory effect of OLE against the human CWR-R1ca, DU-145, PC-3, and LNCaP PC cells (Figure 6A,B). OLE treatments at different concentrations significantly reduced the migration of the four PC cell lines in a dose-dependent manner, with IC_50_ values of 23.3 ± 5.1 μM, 32.7 ± 11.4 μM, 53.8 ± 19.6 μM, and 69.5 ± 32.5 μM for CWR-R1ca, DU-145, PC-3, and LNCaP cells, respectively. Multiple OLE concentrations were tested to investigate its ability to suppress the colonization of CWR-R1ca, DU-145, PC-3, and LNCaP cells (Figure 6C,D). OLE treatments potently inhibited colony formation in CWR-R1ca and DU-145 cell lines with IC_50_ values of 19.3 ± 3.7 and 21.7 ± 6.2 μM, respectively, showing moderate colony formation inhibition against the PC-3 cell line with an IC_50_ value of 35.1 ± 10.4 μM, while OLE was least potent against the LNCaP cell line, recording an IC_50_ of 88.5 ± 19.3 μM.

### 3.5. OLE Suppressed PCSK9 and Normalized LDLR Expression in CWR-R1ca mCRPC Cells

**Figure 7:**
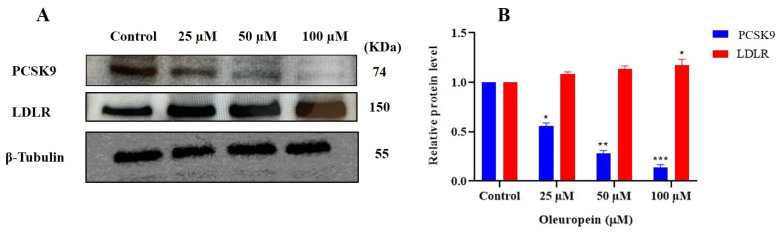
In vitro effects of OLE treatments on the expression levels of PCSK9 and LDLR in CWR-R1ca mCRPC cell line. (**A**) Western blots of PCSK9 and LDLR expression levels in cultured CWR-R1ca cells subjected to 25, 50, and 100 µM of OLE treatments in comparison to VC-treated cells. (**B**) Densitometric analysis of PCSK9 and LDLR expression and the integrated optical density of each band normalized with the corresponding density found for β-tubulin in the same blot. Vertical bars in the graph indicate the normalized integrated optical density of bands visualized in each lane. * *p* < 0.05 and ** *p* < 0.01, and *** *p* < 0.001 indicate statistical significance compared to their respective vehicle-treated controls.

The treatment of CWR-R1ca cells with OLE at 25, 50, and 100 µM significantly suppressed the in vitro expression of PCSK9 and normalized the LDLR expression in a dose-dependent manner (Figure 7A,B). PCSK9 expression levels were downregulated by 43.7%, 71.5%, and 86.4% when the CWR-R1ca cells were subjected to 25, 50, and 100 µM OLE treatments, respectively, in comparison to 100% expression in vehicle control (VC)-treated cells. Meanwhile, OLE 25, 50, and 100 µM treatments resulted in 107.4%, 111.1%, and 114.8% LDLR overexpression levels, respectively, compared to 100% expression in the VC-treated cells.

### 3.6. Effect of OLE on the mCRPC CWR-R1ca-Luc Cells Tumor Progression in a Nude Mouse Xenograft Model

**Figure 8:**
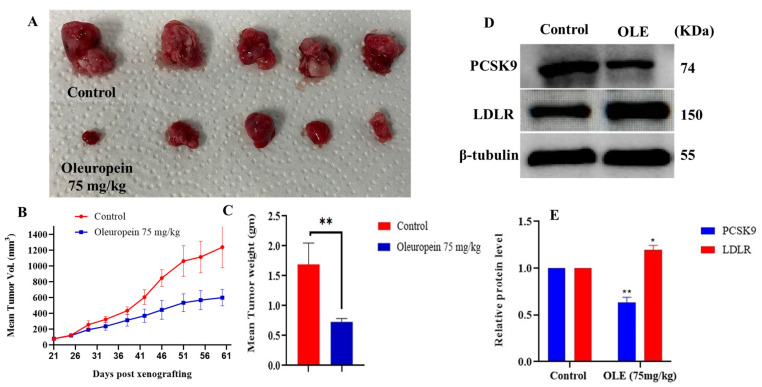
In vivo mCRPC CWR-R1ca-Luc progression suppressive effect of OLE 75 mg/kg, ip 3×/week, versus VC in a nude mouse xenograft model. (**A**) Photographic comparison of surgically excised primary tumors. Top row shows VC-treated primary tumors. Bottom row OLE-treated primary tumors. (**B**) Comparative monitoring of tumors volume in OLE and VC-treated groups over the dosing period. (**C**) Comparison of OLE and VC-treated tumor weights. (**D**) Western blotting image comparison of the effects of OLE versus VC treatments on the expression of PCSK9 and LDLR in collected CWR-R1ca-Luc primary tumors. (**E**) Densitometric analysis of PCSK9 and LDLR expression levels in primary tumors treated with OLE versus vehicle controls. Vertical bars in the graph indicate the normalized integrated optical density of bands visualized in each lane. * *p* < 0.05 and ** *p* < 0.01 indicates statistical significance compared to their respective vehicle-treated controls.

Treatments of male athymic nude mice bearing 50 mm^3^ CWR-R1ca-Luc xenografted tumors (*n* = 5) with OLE 75 mg/kg body weight, ip, 3×/week over a period of 8 weeks, resulted in a significant reduction in the mean tumor volume by 61.5% and the mean tumor weight by 60.1%, in comparison to the vehicle control-treated group (Figure 8A–C). Analogous to the in vitro results, the Western blotting analysis of the collected primary tumor lysates revealed the significant downregulation of PCSK9 (OLE 73.5% versus 100% VC) and upregulation of LDLR in OLE-treated group versus VC-treated mouse tumors (OLE 114.3% versus 100% VC, Figure 8D,E).

### 3.7. Effects of OLE on CWR-R1ca-Luc Cells Locoregional and Distant Tumor Recurrence in a Nude Mouse Xenograft Model

**Figure 9:**
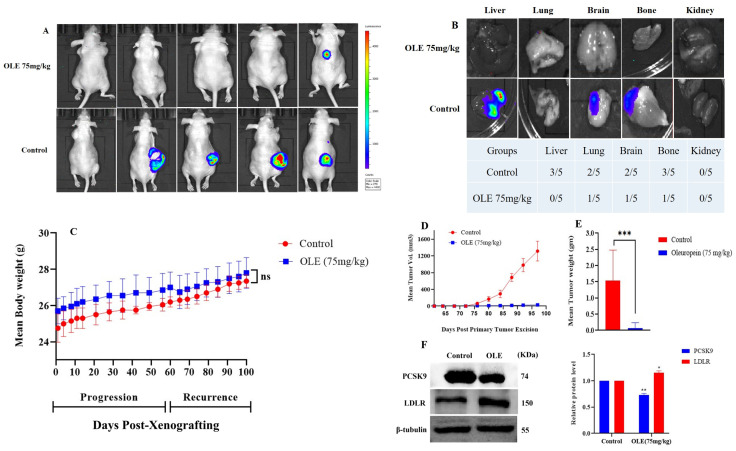
In vivo mCRPC recurrence suppressive effect of OLE 75 mg/kg, ip, 3×/week versus VC in a nude mouse xenograft model after surgical excision of primary tumors. (**A**) Whole animal bioluminescence imaging at the end of the study comparing OLE locoregional recurrence suppressive effects versus VC treatment effects. (**B**) Bioluminescence images of the collected organs (liver, lung, brain bone, kidney) on the last day of the study. (**C**) Mice body weight monitoring throughout the study period. (**D**,**E**) Comparison of OLE versus VC-treated mean tumor volume and weight collected after animals were sacrificed. (**F**) Western blots and densitometric analysis of PCSK9 and LDLR expression levels in OLE versus VC-treated nude mouse livers at the end of the study. Vertical bars in the graph indicate the normalized integrated optical density of bands visualized in each lane. ns indicates statistical non-significance, * *p* < 0.05, ** *p* < 0.01, and *** *p* < 0.001 indicate statistical significance compared to their respective vehicle-treated controls.

Mice used in the previous progression model continued OLE dosing on the next day after the primary tumor surgical excision. OLE was dosed at 75 mg/kg, ip, 3×/week, versus VC for an additional 6 weeks. The OLE treatment prevented locoregional recurrence in four mice out of five, unlike VC-treated mice, which showed four out of five locoregional recurrence (Figure 9A). The first locoregional recurrence tumors in the VC-treated mice group started between 12 and 20 days post-primary tumor excision, unlike the OLE-treated mice group, which showed a single locoregional recurrence after 22 days of surgical excision. This indicated that the OLE treatment induced a 10-day tumor recurrence latency compared to the VC-treated group (Figure 9A). Mice were sacrificed at the end of the experiment, and the organs were harvested and imaged for detecting bioluminescent distant recurrences. The VC-treated group (*n* = 5) exhibited distant recurrences in three livers, three kidneys, two lungs, two brains, and three bones. The OLE-treated mice group (*n* = 5) showed distant recurrences in one lung, one brain, and one bone (Figure 9B). OLE prevented distant recurrences in the liver and kidney. Throughout the study duration, the average animal body weights for the OLE treatment did not differ significantly from the VC treatment (Figure 9C). Drastic 97.6% and 95.3% mean tumor volume and weight reductions, respectively, were observed in the OLE-treated group versus the VC group (Figure 9D,E). The Western blot analysis of collected nude mice liver samples showed that OLE treatments significantly suppressed the PCSK9 expression (65.7%), while simultaneously upregulated LDLR expression (120%) in the treatment group compared to 100% in VC-treated group (Figure 9F).

### 3.8. Comparison of Differentially Expressed Genes (DEGs) in Collected Nude Mouse Xenograft Model Tumors for OLE Treated Versus VC Recurrence

The differential gene expression obtained from the RNA sequencing analysis revealed an altered expression pattern to the PCSK9 and other associated PCSK family members, including PCSK1 and PCSK2, with a recorded fold-change reduction of −1.1, −11.1, and −5.2, respectively, in treated recurred tumors (Table 1). LDLR and LRP1 expression levels increased in primary tumors but only LRP1 increased in recurred tumors.

## 4. Discussion

Prostate cancer (PC) progression and recurrence remain significant clinical challenges, necessitating a multifaceted strategy to effectively intervene, where the currently available approaches that include LH/FSH modulators, anti-AR small molecules, CYP17A1 and CYP27A1 inhibitors, chemotherapies, and immunotherapies are not providing definitive curative outcomes, especially in mCRPC patients, who are susceptible to a high recurrence rate [1,2,35,36]. PC cells rely deeply on androgens for their growth and survival. Through de novo steroidogenesis, mCRPC cells can intracellularly biosynthesize androgens from cholesterol directly [37]. Moreover, PC cells can efficiently uptake cholesterol from the bloodstream for utilization in steroidogenesis [38]. Rising evidence suggests that PC cells can bypass castration therapy by modulating intracrine androgen biosynthesis, enabling the de novo production of androgens within the tumor microenvironment in advanced mCRPC, rendering the ADT no longer competent [39]. The blood lipid profile in PC patients had shown significantly elevated cholesterol and LDL-C levels [40]. Men with hypercholesterolemia are usually at a higher risk to developing high-grade PC [40,41,42]. Nude mice fed on HFD (11% total fat) showed a doubled PC size versus animals fed on a regular chow diet (5% total fat) [16]. The prostate tissues of PC patients have recorded higher membrane and cytoplasmic concentrations of cholesterol and a two-fold increase in nuclear cholesterol when compared to normal counterparts [43].

PCSK9 is a serine protease enzyme mainly produced in the liver, in addition to the brain, intestine, and kidney [6,7,8]. PCSK9 plays a pivotal role in the regulation of cholesterol homeostasis [6,7,8,11,44,45,46]. PCSK9 has shown an aberrant expression pattern in several tumors and has been proved to play an oncogenic role in breast, prostate, colon, gastric, lung, skin, and other cancers [7,10,47]. The mitogenic effects of PCSK9 have been reported through inhibiting tumor cell apoptosis in hepatocellular carcinoma [48]. PCSK9 was found to promote the invasion and migration of gastric cancer cells while inhibiting apoptosis [15]. When PCSK9 was silenced, these mitogenic effects were eliminated, leading to metastasis inhibition [15]. Most current PCSK9-targeting drugs are large molecules, including humanized mAbs and peptidomimetics, which usually fail to cross cellular membranes and cannot act intracellularly. The dual targeting of the PCSK9 expression and its PPI with LDLR by small molecules could effectively inhibit the intracellular PC cholesterol uptake, the key biosynthetic precursor for androgens, and directly target the de novo aberrant PCSK9 dysregulation [16,17]. Research studies have demonstrated that elevated PCSK9 expression is associated with boosted PC cell proliferation, invasion, and migration, proposing its potential mitogenic contribution to tumor progression [17]. Additionally, PCSK9 siRNA therapy significantly promoted cell survival, reduced apoptosis, and shielded the lymph node carcinoma of the prostate (LNCaP) from cell damage by upregulating the expression of cytochrome C (cyto C), B-cell leukemia/lymphoma 2 (Bcl-2), and Bcl-2-associated X protein [49]. A large-scale genetic database offered convincing evidence that the lipid-lowering drugs targeting PCSK9 may restrain the PC incidence [50]. Recent studies indicated that the genetically mediated regulation of PCSK9 is strongly accompanied by a reduced risk of both overall and early onset PC, potentially through a mechanism that implicates lowering lipoprotein (a) levels [51].

The secoiridoid phenolic glucoside (−)-oleuropein (OLE) is the biosynthetic parent of several olive phenolics and is naturally plentiful in unprocessed olive leaves and fruits, which accounts for their bitter taste [52]. OLE has shown several promising pharmacological actions against diabetes and inflammatory disease, with documented antioxidant, antimicrobial, neuroprotective, as well as anticancer effects [53].

This study explored the OLE potential targeting of PCSK9 expression and PPI in mCRPC. In silico modeling generated a PCSK9-LDLR PPI docking model, which was aligned well with the already available X-ray crystal structure of the PCSK9-LDLR complex PDB 3BPS. This model was used to evaluate and compare the ability of OLE and OA to bind at the PCSK9 interface to assess their ability to hinder PCSK9-LDLR binding. Asp374 in PCSK9 is the site of the gain-of-function mutation, and it is always positioned in PCSK9-LDLR crystal structures to interact with the His-306 of LDLR-EGF-A. Thus, Asp374 is validated as the most crucial amino acid to be targeted for interrupting PCSK9-LDLR PPI interactions. A SiteScore of 0.8 has been found to accurately distinguish between drug binding and non-drug-binding sites. Sitemap was able to identify the PCSK9 interface, formed primarily by amino acid residues 367–381 along with the *N*-terminus of the catalytic domain of PCSK9, as possible binding sites for small molecules, with a SiteScore of 0.91 (Figure 2B).

The OLE catechol oxygens contributed hydrogen bonding interactions with Asp374, while the catechol aromatic ring exerted a favorable π-π stacking with the important Phe379 in PCSK9. The OLE sugar moiety extended its interaction with the PCSK9 catalytic domain *N*-terminus residue Ser153 (Figure 3A). The redocking of the PCSK9-OLE complex with LDLR showed a completely different binding pose inaccessible for interaction with LDLR. This inversed docking pose, where EGF-A was bounded from the other side of the PCSK9 structure, showed no interactions with PCSK9 interface or its *N*-terminus regions involved in normal binding with LDLR (Figure 3B). Thus, modelling studies predicted that the OLE binding at the PCSK9 interface and its interactions with crucial amino acid residues Asp374, Phe379, Arg194, and Ser153 might potentially hinder the specific binding of the LDLR EGF-A with the PCSK9 catalytic site.

OLE perfectly filled the PCSK9 interface with an almost 2-fold increase in the binding affinity compared to OA (Figure 3A and Figure 4A). Both OLE and OA were able to interfere with PCSK9-LDLR PPI when the redocking experiments were performed (Figure 3B and Figure 4B). The glucosidic moiety of OLE was hypothesized to form two hydrogen bond interactions with the PCSK9 Asp374, which would achieve greater binding affinity towards PCSK9, unlike the OA, which showed only one hydrogen bond interaction with Asp374, mimicking the His306 substitution in the LDLR EGF-A domain [54]. Previous reports showed that the affinity of PCSK9 binding to the LDLR is enhanced at an acidic pH, suggesting that PCSK9 binds more strongly to LDLR in the lysosomal/endosomal compartments [55]. This has been attributed to the fact that EGF-A–His-306 at a pH of 4.8 is presumably protonated and subsequently forms a salt bridge with the PCSK9 Asp374. Mutation of Asp374 to Tyr would orient the hydroxyl group of tyrosine towards the EGF-A His306, forming a more favorable hydrogen bond interaction, nearly 10-fold more active than the wild-type PCSK9 in mediating the LDLR degradation, due to the 30-fold enhanced PCSK9 binding affinity to LDLR [40].

A diverse panel of PC cells, together with the immortalized human non-tumorigenic RWPE-1 epithelial cell line, were screened against a range of OLE concentrations for effects on their viability. OLE showed modest dose-dependent antiproliferative activities against the mCRPC CWR-R1ca cells, the androgen independent DU-145 cells, the CRPC PC-3 cells, and the androgen-dependent LNCaP cells. Interestingly, OLE only had a negative effect on the non-tumorigenic RWPE-1 epithelial cells at concentrations significantly higher than its cytotoxic levels on PC cells, indicating a high selectivity for malignant cells (Figure 5). Tumor cells migration is a pivotal step in the metastatic cascade. OLE optimally reduced the migration rate of the selected PC cell lines in a dose-dependent manner. OLE remarkably suppressed the PC cell line colony formation in a dose-dependent manner. The mCRPC CWR-R1ca cells were the most sensitive and LNCaP cells the least sensitive in migration and clonogenicity assays. It is noteworthy to mention that the colony formation assay is the in vitro model of choice to mimic the in vivo distant recurrences, since it resembles the clonogenicity of the disseminated tumor cells that escaped the excision surgery, invaded the host–tumor microenvironment to the mouse circulation, and/or resisted the therapeutic regimen (Figure 6). Accordingly, migration and colony formation results elucidate the cytostatic capacity of OLE rather than cytotoxic effects, promoting OLE to inhibit motility and growth without direct effects on survival. The mCRPC cells CWR-R1ca were selected for the in vivo study, owing to their sensitivity in the in vitro assays, dysregulated PCSK9-LDLR axis with high PCSK9 and low LDLR levels [16], and their aggressive-recurrent profile. The CWR-R1ca cells represent an ideal in vivo model to study tumor distant recurrences and metastatic patterns, as this cell line is highly metastatic and expresses wild-type AR, AR-v7, and PSA [41]. OLE in vitro treatments (25–100 µM) successfully reduced the PCSK9 and normalized LDLR expression levels in CWR-R1ca cells (Figure 7).

The previous in vivo testing of OLE used oral and ip routes of administration [42]. The ip route was selected to minimize mice stress by dosing 3× per week instead of 7× per week in oral dosing. A slightly high dose (75 mg/kg, 3×/week) was selected based on the previously reported literature on in vivo OLE dosing, but lower doses should definitely be tested in future. This dose can be translated to 6.1 mg/kg in humans [56]. The experimental nude mice were maintained on HFD throughout the experiment, relying on the previously reported double expression level enhancement with this diet in these cells and the proportional association between HFD and aggressive PC progression [57,58,59]. OLE showed an impressive reduction in primary mCRPC progression, and significantly reduced the tumors weight and volume in comparison to the VC group. This effect had been associated with a reduced PCSK9 expression and a corresponding moderate upregulation in LDLR expression (Figure 8). Tumor recurrence is the root cause of PC patients’ mortality, where the mCRPC is most likely to recur versus other PC phenotypes [60]. A key factor contributing to recurrence is the activation of the dormant tumor cells, which plays a crucial role in creating latency, making metastatic cancer cells highly resistant to both conventional chemotherapy and targeted therapies [61,62]. In the OLE-treated group, minimal locoregional recurrence was observed in only one out of five mice, while in the VC-treated group, gross tumor locoregional recurrences were exhibited in four out of five mice. It is well-supported that every 2.6 adult mouse days are equivalent to one human year [17,63]. Consequently, the 10 day tumor recurrence latency in the OLE-treated group is equivalent to 3.8 years of tumor-free survival in human PC patients. The interaction between the circulating tumor cells and the microenvironment of organs in their surroundings is crucial for distant organ-specific (organotropism) recurrence [64]. Circulating tumor cells migrate to distant organs, form a colony, and establish new tumors when the local microenvironment of the target organ favors successful colonization [64,65]. OLE immensely inhibited locoregional as well as distant tumor recurrences versus VC. This effect had been extended to downregulate PCSK9 and upregulate LDLR expressions in the liver samples of OLE versus VC-treated mice, evidenced by Western blot by the end of the study. The increase in the LDLR level reflects the potential success of OLE in inhibiting PCSK9-LDLR PPI, preventing the PCSK9-driven LDLR degradation. These results validate OLE as a novel small molecule PCSK9 expression and PPI lead suppressor. The non-significant changes in the mean body weight of mice in OLE-treated group and control group at any given time over the progression and recurrence experiments, in addition to its high in vitro selectivity to malignant cells versus non-tumorigenic cells, suggest a high OLE safety profile (Figure 4 and Figure 9).

The mCRPC can transdifferentiate into neuroendocrine prostate cancer (NEPC), an aggressive phenotype with poor prognosis. It had been reported that PCSK1 plays a crucial role in the neuroendocrine system, where PCSK1 is upregulated in NEPC compared to adenocarcinoma samples [66]. PCSK2 was also proved to be a potential neuroendocrine differentiation marker [67]. The downregulation of *PCSK9* mRNA proved to be associated with the downregulation of *PCSK1* and *PCSK2* [66,67]. The extended use of HFD proved to significantly elevate PCSK9 [16], which might justify why the *PCSK9* level was not reduced in OLE-treated primary tumors due to a shorter exposure time to OLE treatment, unlike the case of recurred tumors, which were exposed to OLE treatments for an additional 8 weeks. The CWR-R1ca cells represent an advanced mCRPC phenotype; although it maintained wild and mutant AR expressions, it also expressed all neuroendocrine markers, suggesting its close phenotypic similarity to NEPC [16,22]. OLE-treated recurrence tumors showed a significant downregulation of both *PCSK1* and *PCSK2*, justifying its recurrence-suppressive efficacy against the CWR-R1ca tumors (Table 1). The LDLR and LDL receptor-related protein 1 (LRP1) are both downstream substrates for PCSK9 activation [9,10,11,12]. Both *LDLR* and *LRP1* were slightly upregulated in OLE-treated primary tumors, suggesting the potential interference of OLE with their PPI and PCSK9 catalytic site. Although *LRP1* was slightly increased in OLE-treated recurrence tumors, *LDLR* slightly decreased, which might be a normalization mechanism caused by the recurrent tumor cells.

## 5. Conclusions

The study presented (−)-oleuropein (OLE), which is the major phenolic secoiridoid glucoside in olive leaves, as a novel small molecule lead for the control of mCRPC progression and recurrence. OLE was able to disrupt the PPI of the PCSK9-LDLR complex in silico. OLE perfectly fits at the shallow PCSK9 interface, forming multiple favorable hydrogen bond interactions with amino acids involved in PCSK9-LDLR binding. OLE successfully controlled the progression of mCRPC in a nude mouse xenograft model and impressively suppressed mCRPC locoregional and distant tumor recurrences. OLE not only managed to suppress the PCSK9 but also extended its downregulatory effects to the potential NEPC biomarkers *PCSK1* and *PCSK2*, suggesting its efficacy for advanced PC phenotypes. OLE is a potential PC recurrence lead suppressor that can be developed as a nutraceutical in the future for PC patient and survivor use to control their disease and prevent its relapse.

## Figures and Tables

**Table 1:** Comparison of the Log^2^ FPKM (Fragments/Kilobase Million) fold-change expression of selected proteins in OLE versus VC-treated primary and recurred locoregional CWR-R1ca-Luc tumors.

Gene Symbol	Primary Tumors	Recurrence Tumors
*PCSK1N*	+1.5	+1.4
*PCSK1*	+1.0	−11.1
*PCSK2*	+576	−5.2
*PCSK4*	+1.5	+1.4
*PCSK5*	+1.1	+1.0
*PCSK6*	−1.2	−1.0
*PCSK7*	−1.2	−1.2
*PCSK9*	+5.7	−1.1
*LDLR*	+1.4	−1.0
*LRP1*	+2.3	+1.9

## Data Availability

All data used to support the findings of this study are available in this publication in the figures, tables, or Supplementary Materials.

## Supplementary Materials

The following supporting information can be downloaded at: https://www.mdpi.com/article/10.3390/nu17091445/s1, Figure S1: Chemical structures of (−)-oleuropein and (−)-oleuropein aglycones; Figure S2: Molecular modeling interactions between PCSK9 and LDLR; Table S1: Summary of important interactions in the PCSK9-LDLR generated model; Figure S3: Collected animal organs showing distant recurrences in 1 out 5 in OLE group versus 5 out of 5 in control group; Figure S4: PCSK9, LDLR and β-tubulin total level expressions in mCRPC CWR-R1ca cells raw Western blot images; Figure S5: PCSK9, LDLR and β-tubulin total level expressions in mCRPC primary tumors raw Western blot images from male nude mouse xenograft model, OLE versus VC; Figure S6: PCSK9, LDLR, and β-tubulin total level expressions raw Western blot images for the mCRPC recurrence phase mice livers comparing the effects of OLE versus VC treatments; Figure S7: Volcano plot demonstrates DEGs analysis in tumors of OLE treatment versus VC; Figure S8: Volcano plot demonstrates DEGs analysis in tumors of OLE recurrence versus VC.

## References

[1] Siegel R.L., Kratzer T.B., Giaquinto A.N., Sung H., Jemal A. (2025). Cancer statistics, 2025. CA Cancer J. Clin..

[2] Wang G., Zhao D., Spring D.J., DePinho R.A. (2018). Genetics and biology of prostate cancer. Genes Dev..

[3] Henríquez I., Roach M., Morgan T.M., Bossi A., Gómez J.A., Abuchaibe O., Couñago F. (2021). Current and emerging therapies for metastatic castration-resistant prostate cancer (mCRPC). Biomedicines.

[4] Giacinti S., Poti G., Roberto M., Macrini S., Bassanelli M., DI Pietro F., Aschelter A.M., Ceribelli A., Ruggeri E.M., Marchetti P. (2018). Molecular basis of drug resistance and insights for new treatment approaches in mCRPC. Anticancer. Res..

[5] Go G.W., Mani A. (2012). Low-density lipoprotein receptor (LDLR) family orchestrates cholesterol homeostasis. Yale J. Biol. Med..

[6] Mahboobnia K., Pirro M., Marini E., Grignani F., Bezsonov E.E., Jamialahmadi T., Sahebkar A. (2021). PCSK9 and cancer: Rethinking the link. Biomed. Pharmacother..

[7] Bhattacharya A., Chowdhury A., Chaudhury K., Shukla P.C. (2021). Proprotein convertase subtilisin/kexin type 9 (PCSK9): A potential multifaceted player in cancer. Biochim. Biophys. Acta Rev. Cancer.

[8] Wang L., Li S., Luo H., Lu Q., Yu S. (2022). PCSK9 promotes the progression and metastasis of colon cancer cells through regulation of EMT and PI3K/AKT signaling in tumor cells and phenotypic polarization of macrophages. J. Exp. Clin. Cancer Res..

[9] Barale C., Melchionda E., Morotti A., Russo I. (2021). PCSK9 Biology and Its Role in Atherothrombosis. Int. J. Mol. Sci..

[10] Oza P.P., Kashfi K. (2023). The evolving landscape of PCSK9 inhibition in cancer. Eur. J. Pharmacol..

[11] Bao X., Liang Y., Chang H., Cai T., Feng B., Gordon K., Zhu Y., Shi H., He Y., Xie L. (2024). Targeting proprotein convertase subtilisin/kexin type 9 (PCSK9): From bench to bedside. Signal Transduct. Targ. Ther..

[12] Seidah N.G., Prat A. (2022). The multifaceted biology of PCSK9. Endocr. Rev..

[13] Momtazi-Borojeni A.A., Nik M.E., Jaafari M.R., Banach M., Sahebkar A. (2019). Effects of immunization against PCSK9 in an experimental model of breast cancer. Arch. Med. Sci..

[14] Gu Y., Lin X., Dong Y., Wood G., Seidah N.G., Werstuck G., Major P., Bonert M., Kapoor A., Tang D. (2023). PCSK9 facilitates melanoma pathogenesis via a network regulating tumor immunity. J. Exp. Clin. Cancer Res..

[15] Xu B., Li S., Fang Y., Zou Y., Song D., Zhang S., Cai Y. (2020). Proprotein convertase subtilisin/kexin type 9 promotes gastric cancer metastasis and suppresses apoptosis by facilitating MAPK signaling pathway through HSP70 up-regulation. Front. Oncol..

[16] Abdelwahed K.S., Siddique A.B., Ebrahim H.Y., Qusa M.H., Mudhish E.A., Rad A.H., Zerfaoui M., Abd Elmageed Z.Y., El Sayed K.A. (2023). Pseurotin A validation as a metastatic castration-resistant prostate cancer recurrence-suppressing lead via PCSK9-LDLR axis modulation. Mar. Drugs.

[17] Abdelwahed K.S., Siddique A.B., Qusa M.H., King J.A., Souid S., Abd Elmageed Z.Y., El Sayed K.A. (2021). PCSK9 axis-targeting pseurotin A as a novel prostate cancer recurrence suppressor lead. ACS Pharmacol. Trans. Sci..

[18] Junkins K., Rodgers M., Phelan S.A. (2023). Oleuropein induces cytotoxicity and peroxiredoxin overexpression in MCF-7 human breast cancer cells. Anticancer Res..

[19] Vogel P., Kasper Machado I., Garavaglia J., Zani V.T., de Souza D., Morelo Dal Bosco S. (2014). Polyphenols benefits of olive leaf (*Olea europaea* L.) to human health. Nutr. Hosp..

[20] Ventriglio A., Sancassiani F., Contu M.P., Latorre M., Di Salvatore M., Fornaro M., Bhugra D. (2020). Mediterranean diet and its benefits on health and mental health: A literature review. Clin. Pract. Epidem. Ment. Health.

[21] Shamshoum H., Vlavcheski F., Tsiani E. (2017). Anticancer effects of oleuropein. BioFactors.

[22] Liu L., Ahn K.S., Shanmugam M.K., Wang H., Shen H., Arfuso F., Chinnathambi A., Alharbi S.A., Chang Y., Sethi G. (2019). Oleuropein induces apoptosis via abrogating NF-κB activation cascade in estrogen receptor-negative breast cancer cells. J. Cell Biochem..

[23] Yılmaz G., Özdemir F. (2024). Novel anti-tumor strategy for breast cancer: Synergistic role of oleuropein with paclitaxel therapeutic in MCF-7 cells. Anti-Cancer Agents Med. Chem..

[24] Blanco E., Silva-Pilipich N., Bocanegra A., Chocarro L., Procopio A., Ausín K., Fernandez-Irigoyen J., Fernández L., Razquin N., Igea A. (2024). Oleuropein-driven reprogramming of the myeloid cell compartment to sensitise tumours to PD-1/PD-L1 blockade strategies. Br. J. Cancer.

[25] Hashemi Sheikhshabani S., Amini-Farsani Z., Rahmati S., Jazaeri A., Mohammadi-Samani M., Asgharzade S. (2021). Oleuropein reduces cisplatin resistance in ovarian cancer by targeting apoptotic pathway regulators. Life Sci..

[26] Melo Ferreira D., Oliveira M., Alves R.C. (2025). A comprehensive review of the antitumor activity of olive compounds: The case of olive oil, pomace, and leaf extracts, phenolic alcohols, secoiridoids, and triterpenes. Antioxidants.

[27] Lu C., Wu C., Ghoreishi D., Chen W., Wang L., Damm W., Ross G.A., Dahlgren M.K., Russell E., Von Bargen C.D. (2021). OPLS4: Improving force field accuracy on challenging regimes of chemical space. J. Chem. Theor. Comput..

[28] Yang A.S., Honig B. (2000). An integrated approach to the analysis and modeling of protein sequences and structures. I. protein structural alignment and a quantitative measure for protein structural distance. J. Mol. Biol..

[29] Halgren T.A. (2009). Identifying and characterizing binding sites and assessing druggability. J. Chem. Inf. Mod..

[30] Siddique A.B., Ebrahim H.Y., Tajmim A., King J.A., Abdelwahed K.S., Abd Elmageed Z.Y., El Sayed K.A. (2022). Oleocanthal attenuates metastatic castration-resistant prostate cancer progression and recurrence by targeting SMYD2. Cancers.

[31] Clark A.J., Fakurnejad S., Ma Q., Hashizume R. (2016). Bioluminescence Imaging of an immunocompetent animal model for glioblastoma. JoVE.

[32] Babraham Bioinformatics, The Babraham Institute, Cambridge, United Kingdom (2010). FastQC. A Quality Control Tool for High Throughput Sequence Data. https://www.bioinformatics.babraham.ac.uk/projects/fastqc/.

[33] Liao Y., Smyth G.K., Shi W. (2014). featureCounts: An efficient general-purpose program for assigning sequence reads to genomic features. Bioinformatics.

[34] Benjamini Y., Hochberg Y. (1995). Controlling the false discovery rate: A practical and powerful approach to multiple testing. J. R. Stat. Soc. Ser. B.

[35] Wasim S., Lee S.Y., Kim J. (2022). Complexities of prostate cancer. Int. J. Mol. Sci..

[36] Kulasegaran T., Oliveira N. (2024). Metastatic castration-resistant prostate cancer: Advances in treatment and symptom management. Curr. Treat. Opt. Oncol..

[37] Dillard P.R., Lin M.F., Khan S.A. (2008). Androgen-independent prostate cancer cells acquire the complete steroidogenic potential of synthesizing testosterone from cholesterol. Mol. Cell Endocr..

[38] Raftopulos N.L., Washaya T.C., Niederprüm A., Egert A., Hakeem-Sanni M.F., Varney B., Aishah A., Georgieva M.L., Olsson E., dos Santos D.Z. (2022). Prostate cancer cell proliferation is influenced by LDL-cholesterol availability and cholesteryl ester turnover. Cancer Metab..

[39] Le T.K., Duong Q.H., Baylot V., Fargette C., Baboudjian M., Colleaux L., Taïeb D., Rocchi P. (2023). Castration-resistant prostate cancer: From uncovered resistance mechanisms to current treatments. Cancers.

[40] Horton J.D., Cohen J.C., Hobbs H.H. (2009). PCSK9: A convertase that coordinates LDL catabolism. J. Lipid Res..

[41] Shourideh M., DePriest A., Mohler J.L., Wilson E.M., Koochekpour S. (2016). Characterization of fibroblast-free CWR-R1ca castration-recurrent prostate cancer cell line. Prostate.

[42] Al Shoyaib A., Archie S.R., Karamyan V.T. (2019). Intraperitoneal route of drug administration: Should it be used in experimental animal studies?. Pharm. Res..

[43] Škara L., Huđek Turković A., Pezelj I., Vrtarić A., Sinčić N., Krušlin B., Ulamec M. (2021). Prostate cancer-focus on cholesterol. Cancers.

[44] Spolitu S., Dai W., Zadroga J.A., Ozcan L. (2019). Proprotein convertase subtilisin/kexin type 9 and lipid metabolism. Curr. Opin. Lipid.

[45] Peterson A.S., Fong L.G., Young S.G. (2008). PCSK9 function and physiology. J. Lipid Res..

[46] Tomlinson B., Patil N.G., Fok M., Lam C.W.K. (2021). Role of PCSK9 inhibitors in patients with familial hypercholesterolemia. Endocrinol. Metab..

[47] Singh A., Kumar P., Sonkar A.B., Gautam A.K., Verma A., Maity B., Tiwari H., Sahoo N.G., Keshari A.K., Yadav S.K. (2023). A comprehensive review on PCSK9 as mechanistic target approach in cancer therapy. Mini Rev. Med. Chem..

[48] Zhang S.Z., Zhu X.D., Feng L.H., Li X.L., Liu X.F., Sun H.C., Tang Z.Y. (2021). PCSK9 promotes tumor growth by inhibiting tumor cell apoptosis in hepatocellular carcinoma. Exp. Hematol. Oncol..

[49] Gan S.S., Ye J.Q., Wang L., Qu F.J., Chu C.M., Tian Y.J., Yang W., Cui X.G. (2017). Inhibition of PCSK9 protects against radiation-induced damage of prostate cancer cells. OncoTargets Ther..

[50] Fang S., Yarmolinsky J., Gill D., Bull C.J., Perks C.M., Davey Smith G., Gaunt T.R., Richardson T.G. (2023). Association between genetically proxied PCSK9 inhibition and prostate cancer risk: A Mendelian randomisation study. PLoS Med..

[51] Kirchhofer D., Burdick D.J., Skelton N.J., Zhang Y., Ultsch M. (2020). Regions of conformational flexibility in the proprotein convertase PCSK9 and design of antagonists for LDL cholesterol lowering. Biochem. Soc. Trans..

[52] Bulotta S., Celano M., Lepore S.M., Montalcini T., Pujia A., Russo D. (2014). Beneficial effects of the olive oil phenolic components oleuropein and hydroxytyrosol: Focus on protection against cardiovascular and metabolic diseases. J. Transl. Med..

[53] Barbaro B., Toietta G., Maggio R., Arciello M., Tarocchi M., Galli A., Balsano C. (2014). Effects of the olive-derived polyphenol oleuropein on human health. Int. J. Mol. Sci..

[54] Ni Y.G., Di Marco S., Condra J.H., Peterson L.B., Wang W., Wang F., Pandit S., Hammond H.A., Rosa R., Cummings R.T. (2011). A PCSK9-binding antibody that structurally mimics the EGF(A) domain of LDL-receptor reduces LDL cholesterol in vivo. J. Lipid Res..

[55] Kwon H.J., Lagace T.A., McNutt M.C., Horton J.D., Deisenhofer J. (2008). Molecular basis for LDL receptor recognition by PCSK9. Proc. Natl. Acad. Sci. USA.

[56] Nair A.B., Jacob S. (2016). A simple practice guide for dose conversion between animals and human. J. Basic Clin. Pharm..

[57] Narita S., Nara T., Sato H., Koizumi A., Huang M., Inoue T., Habuchi T. (2019). Research evidence on high-fat diet-induced prostate cancer development and progression. J. Clin. Med..

[58] Labbé D.P., Zadra G., Yang M., Reyes J.M., Lin C.Y., Cacciatore S., Ebot E.M., Creech A.L., Giunchi F., Fiorentino M. (2019). High-fat diet fuels prostate cancer progression by rewiring the metabolome and amplifying the MYC program. Nat. Commun..

[59] Scheinberg T., Mak B., Butler L., Selth L., Horvath L.G. (2023). Targeting lipid metabolism in metastatic prostate cancer. Ther. Adv. Med. Oncol..

[60] Antonarakis E.S., Carducci M.A., Eisenberger M.A. (2010). Novel targeted therapeutics for metastatic castration-resistant prostate cancer. Cancer Lett..

[61] Muzes G., Sipos F. (2017). Metastatic cell dormancy and re-activation: An overview on series of molecular events critical for cancer relapse. Anti-Cancer Agents Med. Chem..

[62] Damen M.P.F., van Rheenen J., Scheele C. (2021). Targeting dormant tumor cells to prevent cancer recurrence. FEBS J..

[63] Dutta S., Sengupta P. (2016). Men and mice: Relating their ages. Life Sci..

[64] Nguyen D.X., Bos P.D., Massagué J. (2009). Metastasis: From dissemination to organ-specific colonization. Nat. Rev. Cancer.

[65] Zhan Q., Liu B., Situ X., Luo Y., Fu T., Wang Y., Xie Z., Ren L., Zhu Y., He W. (2023). New insights into the correlations between circulating tumor cells and target organ metastasis. Signal Transduct. Targ. Ther..

[66] Cejas P., Xie Y., Font-Tello A., Lim K., Syamala S., Qiu X., Tewari A.K., Shah N., Nguyen H.M., Patel R.A. (2021). Subtype heterogeneity and epigenetic convergence in neuroendocrine prostate cancer. Nat. Commun..

[67] Xie Y., Ning S., Hu J. (2022). Molecular mechanisms of neuroendocrine differentiation in prostate cancer progression. J. Cancer Res. Clin. Oncol..

