# Supplementary material for: (−)-Oleuropein as a Novel Metastatic Castration-Resistant Prostate Cancer Progression and Recurrence Suppressor via Targeting PCSK9-LDLR Axis
Source: Nutrients. 2025 Apr 25;17(9):1445. doi: 10.3390/nu17091445 (PMC12073333; doi:10.3390/nu17091445)
Supplement: Supplementary file 1 [file nutrients-17-01445-s001.zip › nutrients-3569153-supplementary.pdf]

nutrients

MDPI

Supplementary Materials

(-)-Oleuropein As A Novel Metastatic Castration-Resistant Prostate Cancer Progression and Recurrence Suppressor via Targeting PCSK9-LDLR Axis

Nehal A. Ahmed[1], Mohamed M. Mohyeldin[2], Hassan Y. Ebrahim[1], Oliver C. McGehee[1], Md Towhidul Islam Tarun[1] and Khalid A. El Sayed[1,*]

[1]School of Basic Pharmaceutical and Toxicological Sciences, College of Pharmacy, University of Louisiana at Monroe, 1800 Bienville Drive, Monroe, LA 71201, USA; atefkhaledahmedabdn@warhawks.ulm.edu (N.A.A.); hebrahim@vcom.edu (H.Y.E.); mcgeheoc@warhawks.ulm.edu (O.C.M.); tarunmt@warhawks.ulm.edu (M.T.I.T.);

[2]Department of Pharmacognosy, Faculty of Pharmacy, Alexandria University, Alexandria 21521, Egypt. mohamed.mohyeldin@alexu.edu.eg (M.M.M.)

*Correspondence: elsayed@ulm.edu (K.A.E.); Tel.: +1-318-342-1725

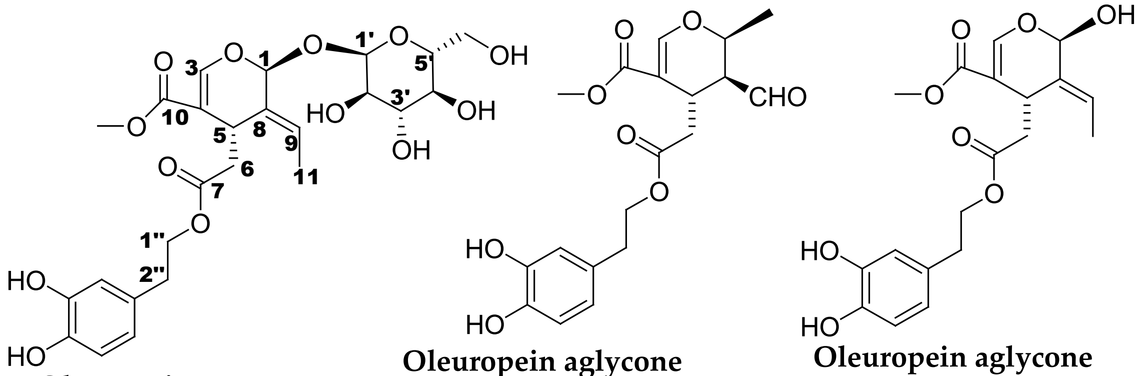

Oleuropein
$C_{25}H_{32}O_{13}$
Exact Mass: 540.1843

Oleuropein aglycone oxidized or aldehydic form
$C_{19}H_{22}O_8$
Exact Mass: 378.1315

Oleuropein aglycone hydroxylic form
$C_{19}H_{22}O_8$
Exact Mass: 378.1315

Figure S1: Chemical structures of (-)-oleuropein and (-)-oleuropein aglycones.

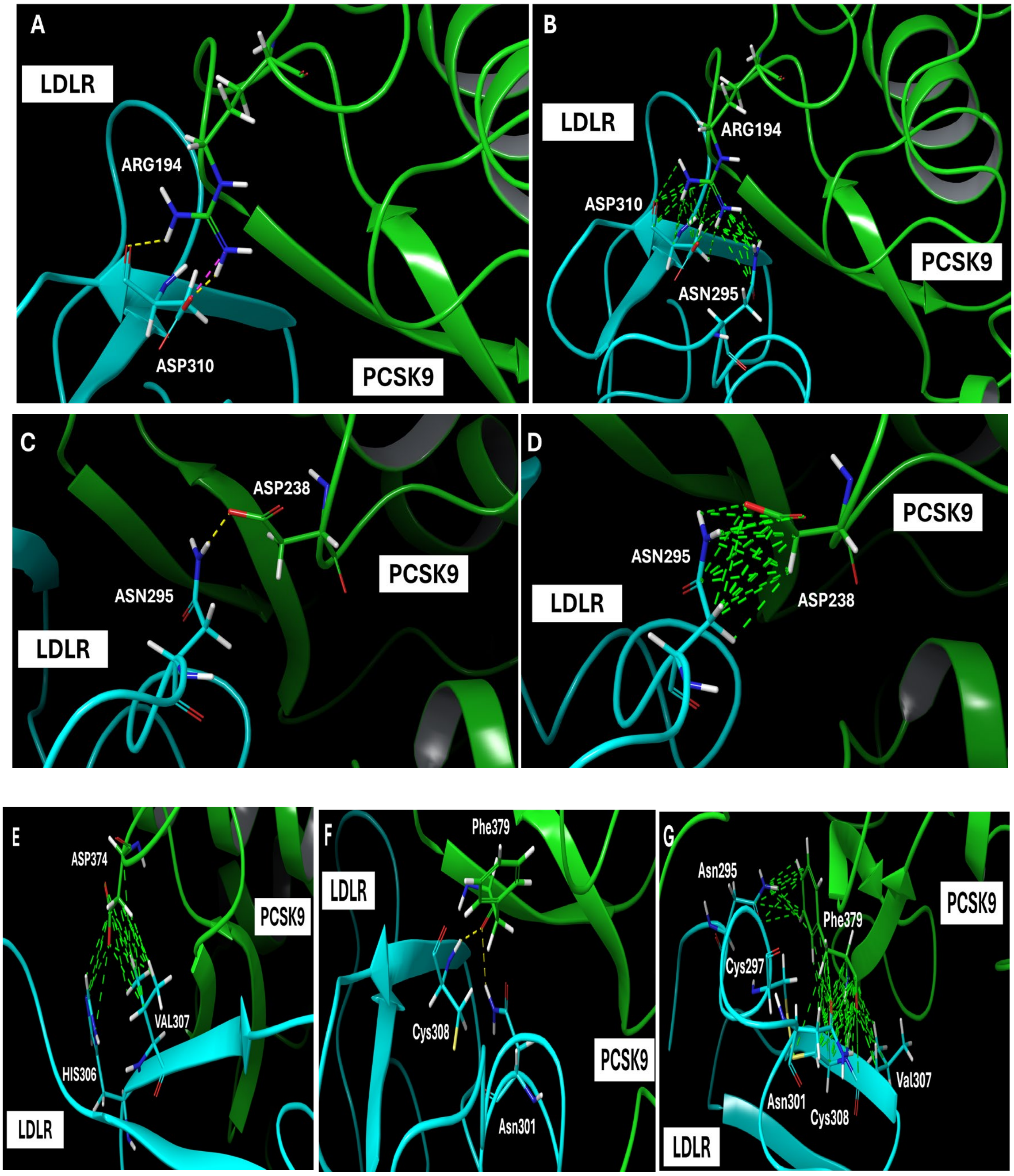

Figure S2: Molecular modeling interactions between PCSK9 and LDLR. (A and B) Key interaction I, (C and D) Key interaction II, (E) Key interaction III and (F and G) Key interaction VI. Hydrogen bonds are shown as yellow dotted lines, amino acids residues interacting on the PCSK9 interface are shown in green and amino acids residues interacting on the LDLR are shown in green.

Table S1: Summary of important interactions in the PCSK9-LDLR generated model. Yellow highlighting denotes key interactions. Green highlighting denotes favorable interactions. A: PCSK9 while E: LDLR. *Van der Waals force, **Hydrogen bond.

Residue	Closest	Distance Å	Specific Interactions	Surface Complementarity	Buried SASA
A:194:Arg	E:310:Asp<br>E:295:Asn	2.1<br>2.8	2x *clash to E:295:Asn<br>2x **hb, 1x salt bridge,<br>4x clash to E:310:Asp	0.59	47.20%
A:238:Asp	E:295:Asn<br>E:298:Leu	2.3<br>3.2	1x hb,<br>1x clash to E:295:Asn	0.88	64.80%
A:369:Ile	E:298:Leu<br>E:297:Cys<br>E:300:Asn<br>E:301:Asn	2.8<br>2.9<br>3.6<br>3.8	1x clash to E:297:Cys	0.78	94.20%
A:372:Ser	E:307:Val	2.6	4x clash to E:307:Val	0.5	54.00%
A:374:Asp	E:307:Val<br>E:306:His	2.4<br>2.5	2x clash to E:306:His<br>6x clash to E:307:Val	0.12	50.80%
A:377:Thr	E:309:Asn<br>E:310:Asp<br>E:308:Cys	1.6<br>2.6<br>3.2	5x clash to E:309:Asn<br>1x hb to E:310:Asp	0.73	59.40%
A:378:Cys	E:307:Val<br>E:308:Cys<br>E:318:Leu<br>E:309:Asn	2.5<br>2.6<br>3.0<br>3.6	9x clash to E:307:Val<br>4x clash to E:308:Cys<br>1x clash to E:309:Asn<br>2x clash to E:318:Leu	0.55	100.00%
A:379:Phe	E:308:Cys<br>E:301:Cys<br>E:307:Val<br>E:295:Asn<br>E:306:His<br>E:297:Cys	2.2<br>2.6<br>3.0<br>3.2<br>3.4<br>3.4	2x clash to E:295:Asn<br>1x clash to E:297:Cys<br>1x hb, 1x clash to E:301:Asn<br>1x clash to E:307:Val<br>1x hb, 5x clash to E:308:Cys	0.72	100.00%
A:380:Val	E:306:His<br>E:301:Asn	3.0<br>3.0	1x clash to E:306:His	0.75	63.30%
E:298:Leu	A:369:Ile<br>A:238:Asp<br>A:155:Pro<br>A:239:Ala<br>A:154:Ile	2.8<br>3.2<br>3.4<br>3.4<br>3.9	3x clash to A:155:Pro	0.87	89.60%

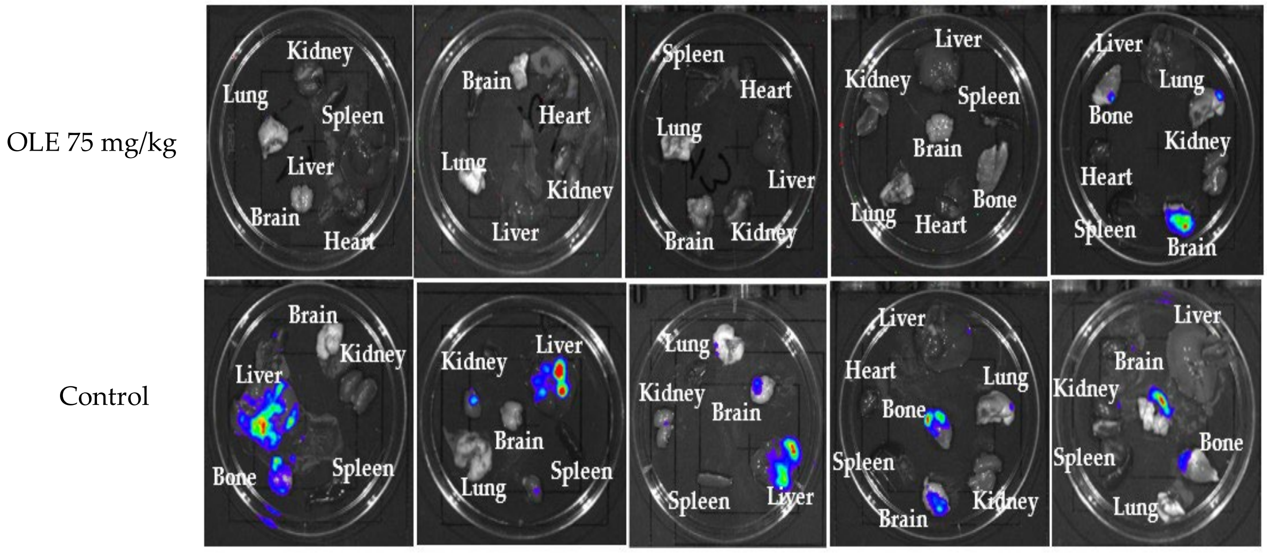

Figure S3: Collected animal organs showing distant recurrences in 1 out 5 in OLE group versus 5 out of 5 in vehicle control group.

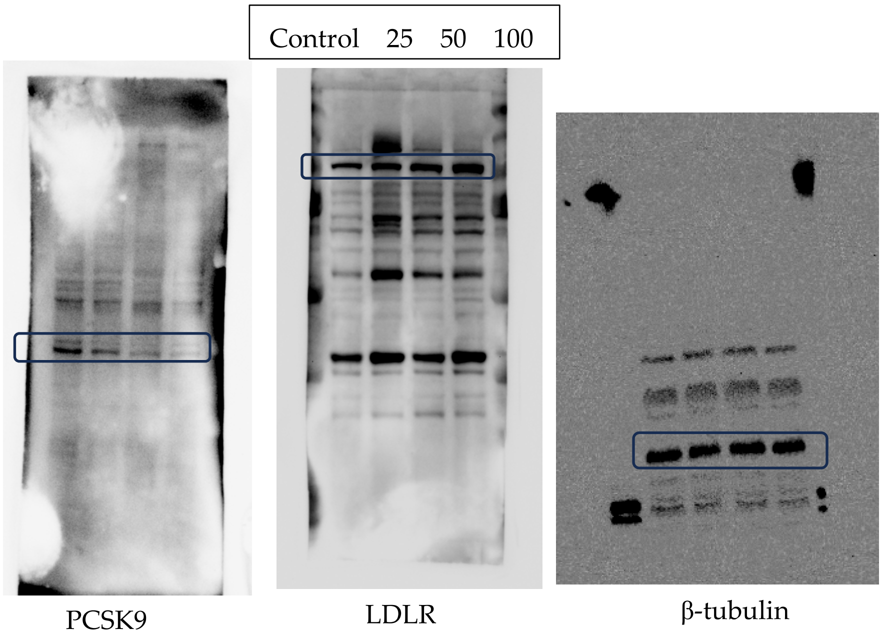

Figure S4: PCSK9, LDLR and β-tubulin total level expressions in mCRPC CWR-R1Ca cells raw Western blot images.

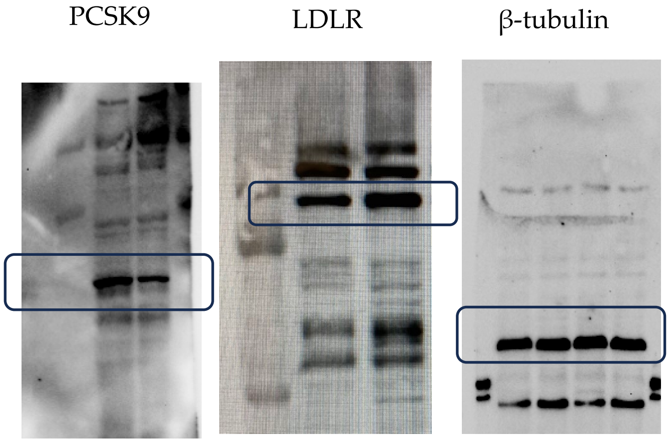

Figure S5*:* PCSK9, LDLR and β-tubulin total level expressions in mCRPC primary tumors raw Western blot images from male nude mouse xenograft model, OLE versus vehicle control.

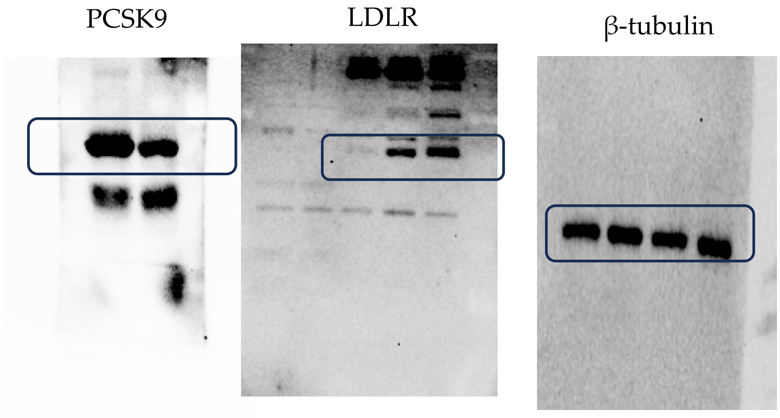

Figure S6: PCSK9, LDLR and β-tubulin total level expressions in mCRPC liver tissues in recurrence phase raw Western blot images from male nude mouse xenograft model, OLE vs vehicle control.

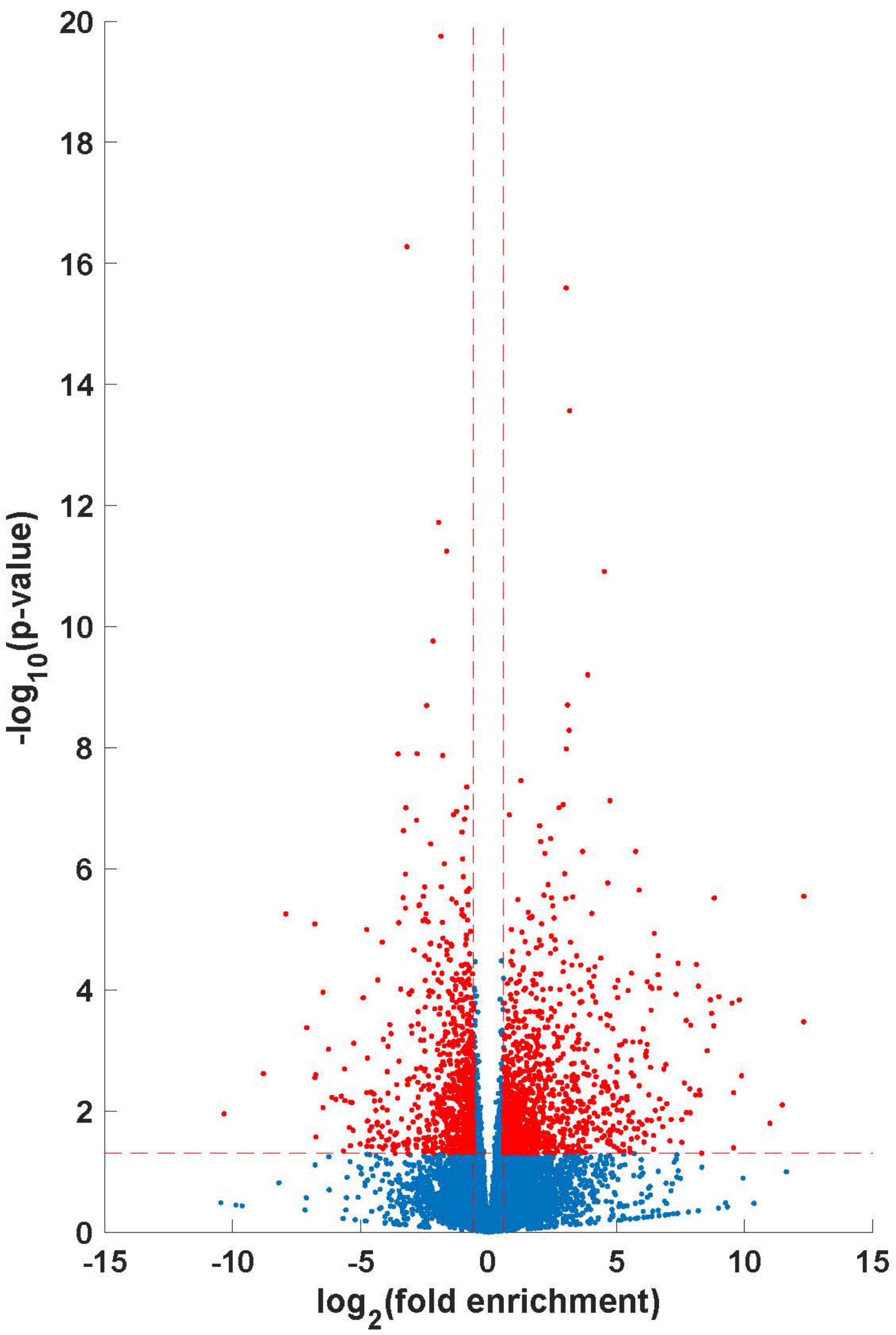

Figure S7: Volcano plot of differentially expressed genes comparing OLE versus VC treatments effects on collected primary mCRPC tumors. Significantly adjusted $p < 0.05$ upregulated and downregulated DEGs are shown in red. Non-significantly upregulated and downregulated DEGs are shown in blue. Red vertical lines are log fold-change of -1.5 and 1.5. The red horizontal line is the negative log adjusted *p*-value of 0.05.

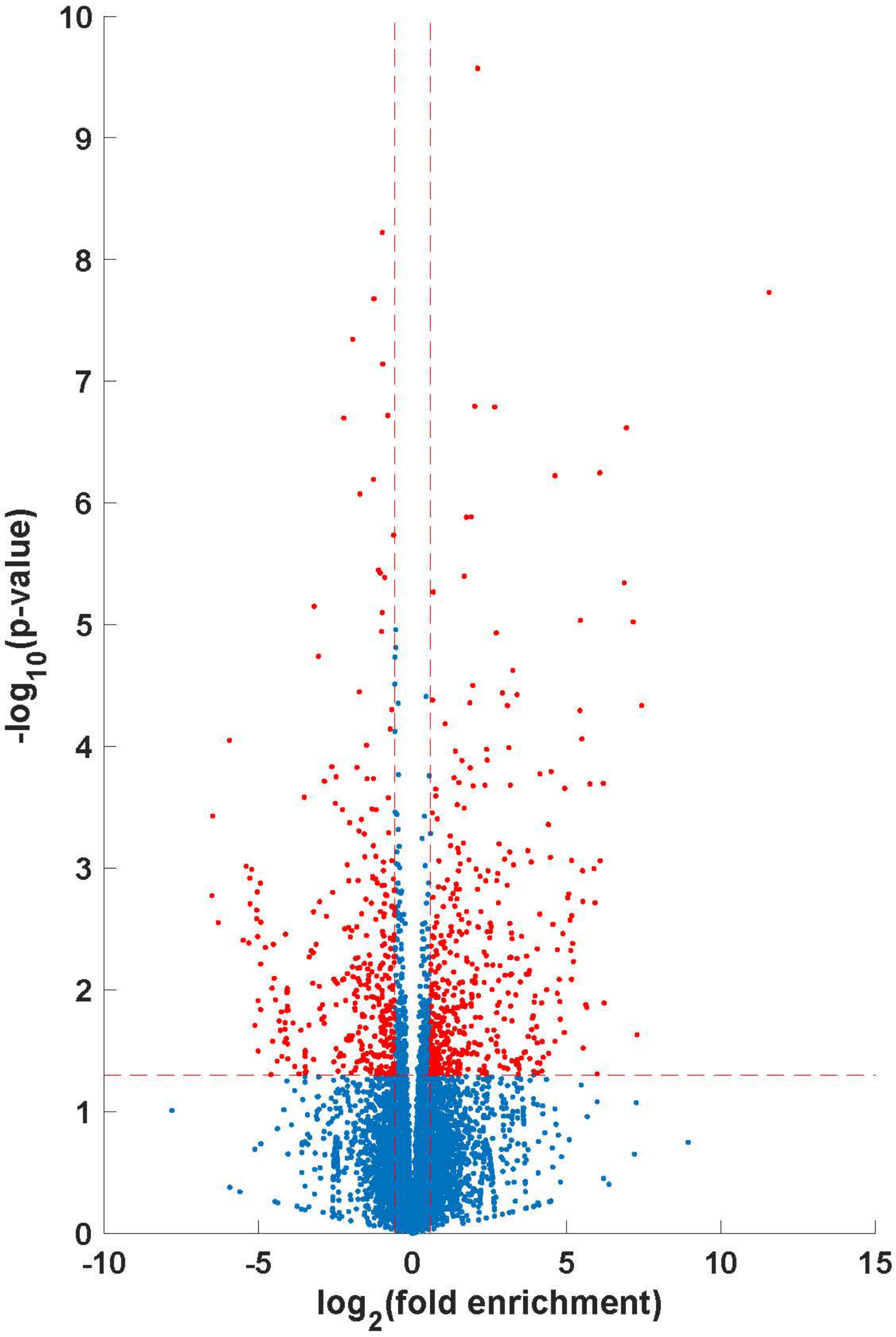

Figure S8: Volcano plot of differentially expressed genes comparing OLE versus VC treatments effects on collected recurrence mCRPC tumors. Significantly adjusted $p < 0.05$ upregulated and downregulated DEGs are shown in red. Non-significantly upregulated and downregulated DEGs are shown in blue. Red vertical lines are log fold-change of -1.5 and 1.5. The red horizontal line is the negative log adjusted p-value of 0.05.
